# *Pseudomonas putida* as saviour for troubled *Synechococcus elongatus* in a synthetic co-culture – interaction studies based on a multi-OMICs approach

**DOI:** 10.1038/s42003-024-06098-5

**Published:** 2024-04-12

**Authors:** Franziska Kratzl, Marlene Urban, Jagroop Pandhal, Mengxun Shi, Chen Meng, Karin Kleigrewe, Andreas Kremling, Katharina Pflüger-Grau

**Affiliations:** 1 Professorship for Systems Biotechnology, TUM School of Engineering and Design, Technical University of Munich, Garching, Germany; 2 Department of Chemical and Biological Engineering, University of Sheffield, Sheffield, United Kingdom; 3 Bavarian Center for Biomolecular Mass Spectrometry (BayBioMS), TUM School of Life Sciences, Technical University of Munich, Freising, Germany

**Keywords:** Industrial microbiology, Industrial microbiology, Microbial communities

## Abstract

In their natural habitats, microbes rarely exist in isolation; instead, they thrive in consortia, where various interactions occur. In this study, a defined synthetic co-culture of the cyanobacterium *S. elongatus cscB*, which supplies sucrose to the heterotrophic *P. putida cscRABY*, is investigated to identify potential interactions. Initial experiments reveal a remarkable growth-promoting effect of the heterotrophic partner on the cyanobacterium, resulting in an up to 80% increase in the growth rate and enhanced photosynthetic capacity. Vice versa, the presence of the cyanobacterium has a neutral effect on *P. putida cscRABY*, highlighting the resilience of pseudomonads against stress and their potential as co-culture partners. Next, a suitable reference process reinforcing the growth-promoting effect is established in a parallel photobioreactor system, which sets the basis for the analysis of the co-culture at the transcriptome, proteome, and metabolome levels. In addition to several moderate changes, including alterations in the metabolism and stress response in both microbes, this comprehensive multi-OMICs approach strongly hints towards the exchange of further molecules beyond the unidirectional feeding with sucrose. Taken together, these findings provide valuable insights into the complex dynamics between both co-culture partners, indicating multi-level interactions, which can be employed for further streamlining of the co-cultivation system.

## Abstract (web summary)

This study investigates interspecies interaction in a synthetic co-culture consisting of the sucrose-secreting cyanobacterium *S. elongatus cscB* and its sucrose-consuming partner *P. putida cscRABY* by employing a comprehensive multi-OMICs approach.

## Introduction

Over the last two decades, a paradigm shift has started in biotechnology, expanding beyond the historically-grown focus on single-species or so-called axenic cultures. This change involves the development of controllable co-cultures comprising two or more species within the same reaction vessel. Considering that microbes barely live separately in nature expands the bioproduction of value-added compounds through novel pathways and strategies. Furthermore, co-cultures enable us to understand synergistic effects and uncover otherwise hidden behaviours, allowing for targeted utilisation of microbial capabilities^1,2^.

The dynamics of a community or a co-culture are determined by interactions of individual cells, which normally act in their local niches. Most microbes have evolved to thrive in the presence of neighbouring species, which can present either potential threats or offer benefits, and they have adapted accordingly^3^. Those interactions can be divided into four general classes: mutualism, neutralism, commensalism, and parasitism^4^. For instance, a mutualistic interaction, where all community members profit, can appear as cross-feeding or syntrophy. Here, one partner does not completely metabolise a given substrate, which then, in turn, is accessible to another partner. The latter might remove toxins or harmful gases, creating the environment needed for the entire community^4^.

The design of a synthetic co-culture is not trivial, as multiple aspects and parameters, such as medium composition, need to be considered^5,6^, and in the best-case scenario, resulting co-cultures should align with a sustainable bioprocess. In most cases, it is intended that the microbes live under the premise of the division of labour, which allows different traits of microbes to complement each other in a profitable way^3,7^. One example of doing so is pairing heterotrophs with phototrophs^8^. This composition of microbes is prevalent in nature, which can be observed in lichens and microbial mats^9^. Here, the photoautotrophic member, such as cyanobacteria or algae, uses solar power to fix CO_2_ into organic carbon, a portion of which then in turn is accessible to the heterotrophic members of the community. In the case of synthetic co-cultures, the heterotrophic partner can be employed to convert the carbon source provided into value-added products by engineered and optimised metabolic pathways. The model organism *Synechococcus* PCC 7942 naturally accumulates sucrose as a compatible solute when exposed to elevated NaCl concentrations. This trait was exploited to construct the sucrose secreting strain *S. elongatus cscB* by genomic integration of the *cscB* gene encoding a H^+^/sucrose symporter^10^. The resulting strain secretes sucrose into the surrounding medium with a rate of up to 28 mg L^−1^ per hour^10,11^. Up to now, several robust synthetic co-cultures have been constructed employing *S. elongatus cscB*, and valuable compounds like α-amylase, polyhydroxyalkanoates (PHAs), or isoprene have been successfully produced^11–13^. The metabolic capacity of the co-culture processes could be expanded depending on the heterotrophic partner used, such as *Escherichia coli*, *Pseudomonas putida*, *Bacillus subtilis*, or *Saccharomyces cerevisiae*.

In contrast to natural communities that are highly complex structures with overlaying interactions, synthetic co-cultures are very well suited to study interactions between and within the involved species^5^. The inherent definition of a synthetic defined co-culture is that the partners have not evolved together or, at least, that the connection between them has not evolved driven by nature. Therefore, we assume that non-engineered feedback and/or communication between the partner organisms have their origin in a general answer adopted from their individual natural habitats. In this study, we set out to investigate those non-engineered interactions that might guide us towards a better understanding of synthetic co-cultures in general and how to design them. To this end, we used a synthetic co-culture consisting of the cyanobacterium *S. elongatus cscB* and the soil-bacterium *P. putida cscRABY*, which was recently employed for PHA production in our lab^12^. In this co-culture, *P. putida cscRABY* has been engineered to transport and metabolise sucrose by the integration of the *cscRABY* operon into the chromosome^14^. In the work described here, first, a suitable reference experiment was set up, which allowed us to compare the co-culture with the respective axenic cultures of *S. elongatus cscB* or *P. putida cscRABY* to identify and investigate the putative interaction of the co-culture partners with each other. Furthermore, we set out to analyse the co-culture not only on a physiological level but also on the transcriptome, proteome, and metabolome level by employing a multi-OMICs approach, which has been demonstrated to be a powerful tool to decipher hidden traits of synthetic communities^13,15^.

## Results and discussion

### Influence of the co-culture partners on each other’s growth

**Fig. 1:**
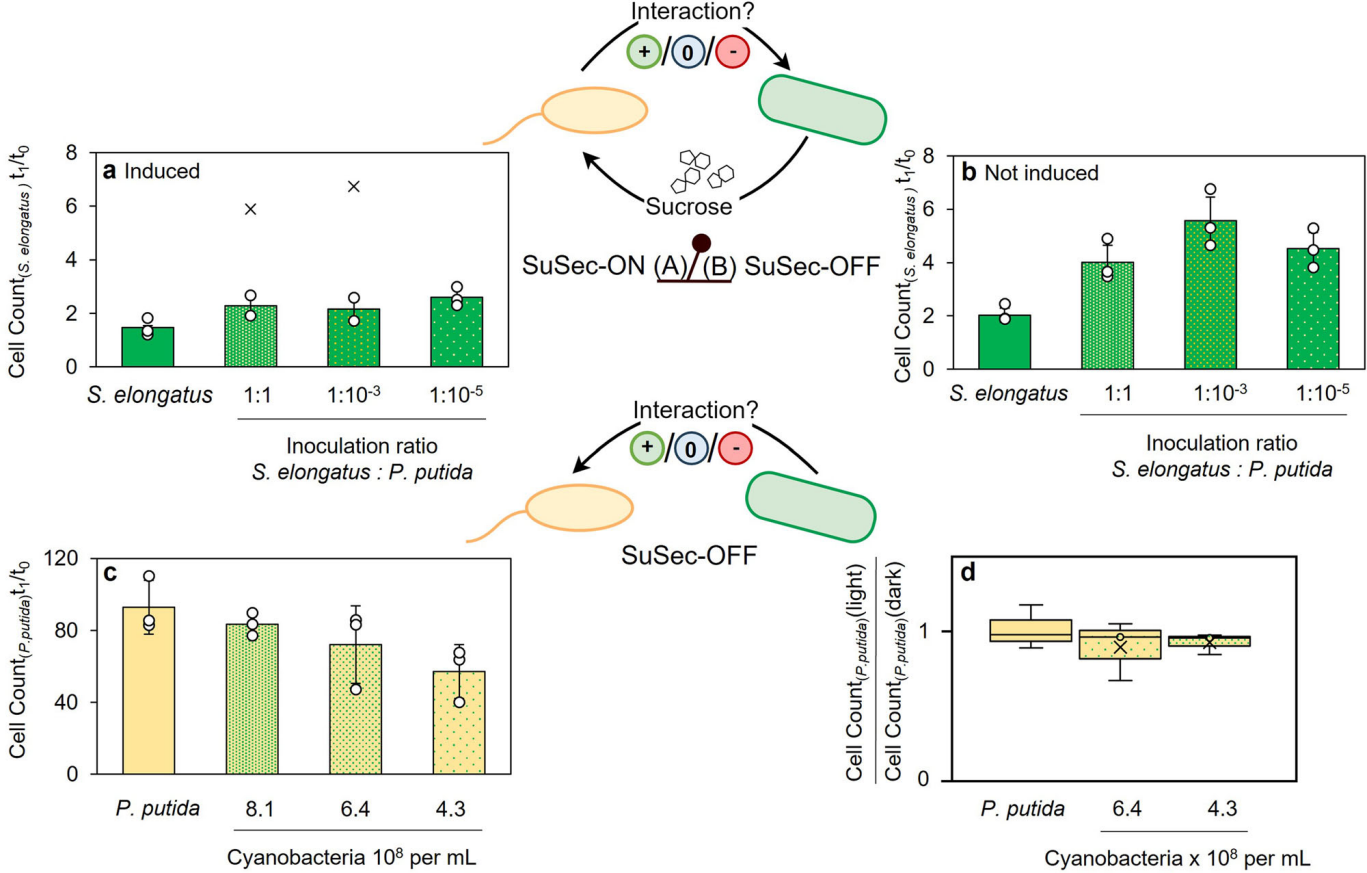
Physiological influence of the co-culture partners on each other’s growth. **a** Influence of the heterotrophic partner on the cell count of *S. elongatus cscB* in axenic culture and in different co-cultures with decreasing *P. putida cscRABY* inoculation density after 24 h when sucrose secretion is induced (SuSec-ON status) or **b** when the growth of the heterotrophic partner is supported by an external sucrose batch (SuSec-OFF status). Data is normalised to the start cell count (t_0_). **c** Influence of the phototrophic partner on the cell count of *P. putida cscRABY* after 24 h of growth in axenic culture or in three co-cultures with decreasing *S. elongatus cscB* inoculation density in the SuSec-OFF situation. Data is normalised to the start cell count (t_0_). **d** Comparison of *P. putida cscRABY* cell count after 24 h grown in light and dark, as axenic-culture and in two co-cultures with different cyanobacterial inoculation cell counts in the SuSec-OFF situation. *Experimental conditions*: 12-well plates with 1.6 ml BG11^+^ supplemented with 150 mM NaCl, 25 °C (or 30 °C), 120 rpm, 22 µE or darkness and no additional aeration with the addition of 0.1 mM IPTG (for **a**) or 1 g L^−1^ sucrose (for **b**–**d**). In all cases, data is derived from *n* *=* 3 biologically independent experiments (individual data points shown as circles, x represent outliers), and error bars represent the standard deviation (based on a sample) of the replicates, calculated using the *n* *−* 1 method.

It was frequently observed that cyanobacteria grow more efficiently in co-cultivation with heterotrophic bacteria both in natural^9,16^ and in synthetic co-cultures^11^. To this end, we investigated the growth of both strains in the co-culture compared to the axenic cultures in different scales and conditions (see Supplementary Note S1 and Supplementary Fig. S1). To analyse the influence of *P. putida cscRABY* on the initial growth of *S. elongatus cscB* in 12-well plates at a 1.6 mL scale, we differentiated between a “SuSec-ON” and a “SuSec-OFF” status of the synthetic connection, brought about by the inducible exchange of sucrose (Fig. 1a, b). In the SuSec-ON situation, the sucrose secretion by *S. elongatus cscB* is induced, which is not the case in the SuSec-OFF situation. Here, an additional batch of 1 g L^−1^ sucrose was added to all cultures, including the axenically grown *S. elongatus cscB*, to support heterotrophic growth and to identify effects independent of the synthetic connection. To investigate the influence of different inoculation ratios (phototroph:heterotroph), the co-culture was inoculated with varying amounts of *P. putida cscRABY* to reach *S. elongatus* to *P. putida* cell ratios of 1:1, 1:10^−3^, and 1:10^−5^ and after 24 h cell counts of both strains were determined.

As shown in Fig. 1, in all co-cultures, cyanobacterial cell counts were higher compared to the axenic culture, suggesting that the presence of *P. putida cscRABY* promotes the initial growth of *S. elongatus cscB* (left bar in Fig. 1a, b). This effect was independent of whether the synthetic connection via the sucrose feed (SuSec) was ON or OFF; however, it was more pronounced in the SuSec-OFF situation. Higher cyanobacterial cell counts are reached in the SuSec-OFF case due to a general reduction of growth when sucrose secretion is induced. An influence of the inoculation ratio can only be observed in the SuSec-OFF situation, where the positive impact of the presence of *P. putida cscRABY* on the initial growth of the cyanobacterium was less pronounced at an inoculation ratio of 1:1 compared to the situation with fewer *P. putida cscRABY* cells. A possible explanation might be that the higher *P. putida cscRABY* cell densities reached within these 24 h caused a more substantial shading on *S. elongatus cscB*, reducing light availability for photosynthesis in the cyanobacterium.

In a co-culture study by Hays et al., *S. elongatus cscB* was found to have a significant negative impact on various heterotrophs, particularly on the gram-positive bacterium *Bacillus subtilis*^11^. In contrast to this, we could not identify an apparent effect of high densities of *S. elongatus cscB* on the growth of *P. putida cscRABY* within 24 h, and, if any, there might be a tendency towards weaker growth of *P. putida cscRABY* when inoculated with less of cyanobacterial cells (Fig. 1c). In previous studies, reactive oxidative species (ROS: ${{{{{{{\rm{O}}}}}}}_{2}}^{-}$, ${{{{{{\rm{OH}}}}}}}^{\cdot }$, ${{{{{{\rm{H}}}}}}}_{2}{{{{{{\rm{O}}}}}}}_{2}$) produced by *S. elongatus cscB* were shown to be the most invasive substances for heterotrophic growth^11,13,17^. Thus, as ROS are side products of photosynthesis, we performed the co-cultivations with or without light in 12-well plates at 1.6 mL scale, and compared them to axenic cultures of *P. putida cscRABY* grown under equal conditions. Two different cyanobacterial cell densities were used for inoculation in the co-culture, as shown in Fig. 1d. Still, no statistically significant difference in the heterotrophic growth could be detected (unpaired T-test, α = 0.05). Therefore, the formation of ROS through photosynthesis had no detectable influence on the growth of *P. putida cscRABY* under the conditions tested.

### Influence of illumination, induction, and inoculation time of *P. putida cscRABY* on *S. elongatus cscB*

**Fig. 2:**
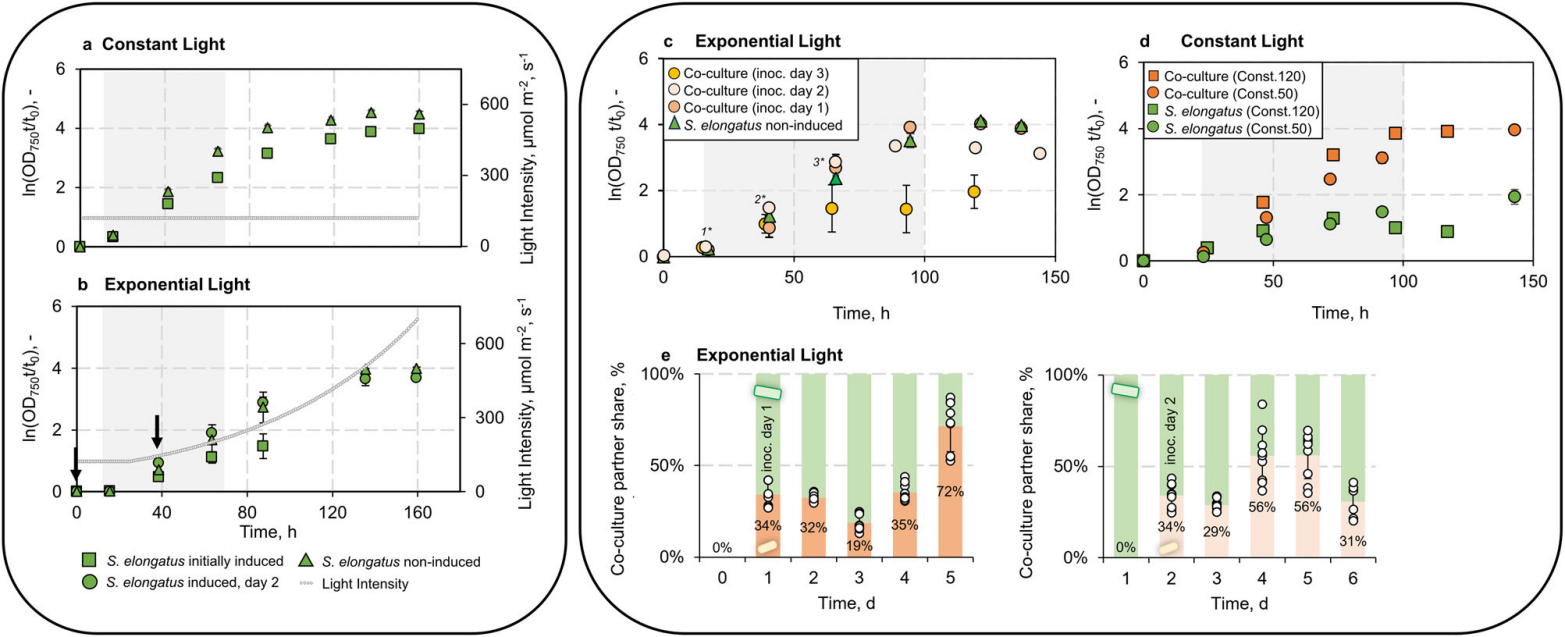
Influence of induction of sucrose secretion and of the illumination profile. **a** Axenic growth of *S. elongatus cscB* with different time points of induction of the *cscB* expression (non-induced, initially induced, or induction after two days) with constant light at 150 µmol m^−2^ s^−2^ and **b** with an exponential light setting. With an exponential light profile, initial induction of sucrose secretion led to photobleaching and the cultivation was stopped after ~90 h. The grey area indicates the data points used to calculate the growth rates (Supplementary Note S2). The arrows mark the time points of induction. **c** Growth of axenic *S. elongatus cscB* with and without induction of *cscB* expression, and of the co-cultures inoculated with *P. putida* *cscRABY* after 16 h (1* = day 1), 46 h (2* = day 2), or after 65 h (3* = day 3) under constant light conditions and **d** with an exponential light setting. The grey area indicates the data points used for calculating the growth rates shown in *Table* *1*. **e** Ratio of phototroph:heterotroph over time with an exponential light profile and inoculation of *P. putida cscRABY* to the co-culture at day 1 or day 2. Individual data points are represented by white circles. *Experimental conditions* **a**, **b**, **e**: BG11^+^ supplemented with 150 NaCl and 0.1 mM IPTG, when *cscB* expression was induced. Constant light: 150 µmol m^−2^ s^−2^ for 160 h; Exponential light: 24 h at 120 µmol m^−2^ s^−2^ followed by an exponential rising with a doubling time t_d_ *=* 52 h. *Experimental conditions* **c**, **d**: 25.5–34 °C, 2% CO_2_, BG11^+^ + 150 mM NaCl, volume 95 mL; Const.120 = constant light profile with 120 µmol m^−2^ s^−2^ and Const.50 = constant light profile with 50 µmol m^−2^ s^−2^, exponential light: 120 µmol m^−2^ s^−2^ constant for 24 h followed by exponential rising with t_d_ *=* 52 h. Data is derived from *n* *=* 3 biologically independent experiments, and error bars represent the standard deviation (based on a sample) of the replicates, calculated using the *n* *−* 1 method.

To analyse the co-culture in more detail, we switched the cultivation to the HD-9.100 CellDeg platform system, which permits parallel co-cultivations under comparable conditions and ensures high reproducibility. We started by analysing the effect of the illumination profile, the induction of sucrose secretion, and the time point of induction on the growth of *S. elongatus cscB* (Fig. 2). With a constant illumination of 150 µmol m^−2^ s^−1^ and without IPTG induction, *S. elongatus cscB* grew with a rate of 0.064 ± 0.001 h^−1^. The presence of 0.1 mM IPTG in the culture, however, nearly halved the growth rate to 0.036 ± 0.004 h^−1^ (Fig. 2a and Supplementary Note S2). This effect is not due to negative feedback of the sucrose accumulated in the medium, as confirmed by growing the cells in the presence of sucrose (Supplementary Note S3 and Supplementary Fig. S2), but rather suggests a rechannelling of the fixed carbon into sucrose secretion instead of biomass formation. This is in accordance with previous studies, where it was reported that induction of the CscB symporter reduces the biomass accumulation of *S. elongatus cscB* while increasing the total carbon fixation^10,18^.

Next, we analysed the influence of an exponential light profile on the cyanobacterial growth behaviour (Fig. 2b). As observed with the constant illumination, induction of the sucrose secretion resulted in a decreased growth rate (Supplementary Table S1). However, here, the effect of induction was way more severe than in conditions of constant light, as cultures did not just have reduced growth rates but also went into a state of photobleaching manifested as visible pigment loss after 80 h at ~300 µmol m^−2^ s^−1^. By shifting the induction to day 2, instead of adding the inducer from the beginning of the cultivation, this effect could be avoided, and growth rates reverted to what was observed in the absence of IPTG (Supplementary Table S1). Furthermore, a higher amount of sucrose could be measured in the supernatant of the culture, in which *cscB* expression was induced on day 2. These cultures accumulated three times more sucrose after 63.5 h than those growing from the beginning in the presence of the inducer. This trend persisted, and after 87.5 h, the level of sucrose accumulated had risen to 0.75 ± 0.38 g L^−1^, while the cultures grown in the presence of IPTG from the beginning exhibited only a slight increase (see Supplementary Note S4 and Supplementary Fig. S3). Taken together, the interplay between the time point of induction and the illumination profile emerged as a significant factor influencing the growth behaviour of the cyanobacterium. We hypothesise that a prolonged phase after inoculation without induction of *cscB* expression facilitated a better adaptation of *S. elongatus cscB* to the environmental conditions^19^. This photoacclimatisation, in turn, could lead to notable differences in photosynthetic activity, resulting in enhanced growth, increased sucrose accumulation, and protection against photobleaching.

After analysing the cyanobacterial growth in axenic cultures, we set out to study the co-cultivation with *P. putida cscRABY*. We observed that under the detrimental conditions of an exponential light profile and initial induction of sucrose secretion, the presence of *P. putida cscRABY* in the co-culture rescued *cscB*-expressing *S. elongatus cscB* from photobleaching and also led to a higher cyanobacterial growth rate (Fig. 2c). Additionally, the time point of inoculation of *P. putida cscRABY* had an influence on the growth behaviour and growth rate of *S. elongatus cscB*. The addition of *P. putida cscRABY* to the culture within the first 50 h (inoc. day 1, inoc. day 2) had a positive effect on the growth of the cyanobacterium. For instance, when *P. putida cscRABY* was inoculated on day 1, *S. elongatus cscB* exhibited a 32% higher growth rate in comparison to the cultures not expressing *cscB* and a 61% higher growth rate compared to the cyanobacterial cells expressing *cscB* (Table 1). Inoculation on day three, however, could no longer restore the growth behaviour to the one observed with *S. elongatus cscB* grown without IPTG.

**Table 1:** Growth rates of *S. elongatus cscB* and *P. putida cscRABY* in axenic cultures and co-cultures with constant illumination (Const.120: 120 µmol m^−2^ s^−2^, Const.50: 50 µmol m^−2^ s^−2^) or an exponential light profile (Expo.) and different inoculation time points (Inoc.) of the heterotrophic partner; mean and standard deviation are derived from three different cultures cultivated in parallel

Condition		*S. elongatus* growth rate, h^-1^		
Light	Inoc.	Non-induced	Axenic, induced	Co-culture
Const.120	Day 1	n.p.^a^	0.027 ± 0.006	0.065 ± 0.005
Const.50	Day 1	0.020 ± 0.003	0.010 ± 0.001	0.056 ± 0.003
Expo.	Day 1	0.057 ± 0.002	0.033 ± 0.001	0.084 ± 0.001
Expo.	Day 2	0.055 ± 0.002	0.044 ± 0.006	0.066 ± 0.02
Expo.	Day 3	0.051 ± 0.002	0.031 ± 0.006	0.046 ± 0.002

^a^Not performed.

These observations prompted the question of why the presence of *P. putida cscRABY* has a growth-promoting effect on *S. elongatus cscB*. One possible explanation could be the physical protection from high light intensities. For *S. elongatus* PCC 7942, the parental strain of the derivative used in this study, light intensities higher than 400 µmol m^−2^ s^−1^ are already considered as high light and intensities of 200 µmol m^−2^ s^−1^ can induce oxidative stress, which is noticeably lower than for other cyanobacteria^20,21^. To test this hypothesis, we chose two non-harmful constant light intensities, 50 µmol m^−2^ s^−1^ (low) and 120 µmol m^−2^ s^−1^ (medium), and compared the growth of *S. elongatus cscB* in axenic culture to that in the co-culture (Fig. 2d). We observed a similar growth-promoting effect of the presence of the co-culture partner, as described above with the exponential light profile, even though the presence of the heterotrophic partner under these low light conditions should rather hamper the growth of the cyanobacterium due to shading from light. For instance, with a constant illumination of 50 µmol m^−2^ s^−1^, the presence of *P. putida cscRABY* prevented *S. elongatus cscB* from an early entry into the stationary phase and increased its growth rate by 64% compared to the cells not expressing *cscB* and even by 82% compared to cells expressing *cscB* (Table 1). The same trend was observed with a medium light intensity. From these experiments, we concluded that the growth-promoting effect of the presence of *P. putida cscRABY* on *S. elongatus cscB* cannot be reduced to the mere protection from high light intensities but rather is the result of a more complex interplay of different factors. As *S. elongatus cscB* grown under non-inducing conditions was not drastically affected by the different light profiles, we assumed that sucrose secretion contributes to the stress perceived by *S. elongatus cscB* that finally leads to photobleaching and reduced growth.

In order to see whether the population ratio provides a hint at the origin of the positive effect on the cyanobacterium, we determined the cell ratio of phototrophic to heterotrophic cells in the co-cultures (Fig. 2e). Although the initial inoculation ratio was the same with 34% of *P. putida cscRABY* to 66% of *S. elongatus cscB*, varying phototroph:heterotroph cell ratios adjusted themselves over time, consequently leading to different shading conditions in each experiment. Nevertheless, the overall behaviour of the co-cultures was comparable irrespective of the time point of addition of the heterotrophic partner (compare Fig. 2c), hinting again towards more complex processes being involved in the growth promoting effect observed by the addition *of P. putida cscRABY*. In order to gain a deeper understanding of these processes and the possible interplay between *P. putida cscRABY* and *S. elongatus cscB*, which extends beyond the mere exchange of sucrose, we chose the exponential light profile for a comprehensive OMICs-driven investigation.

### Co-culture reference experiments for multi-OMICs analysis

**Fig. 3:**
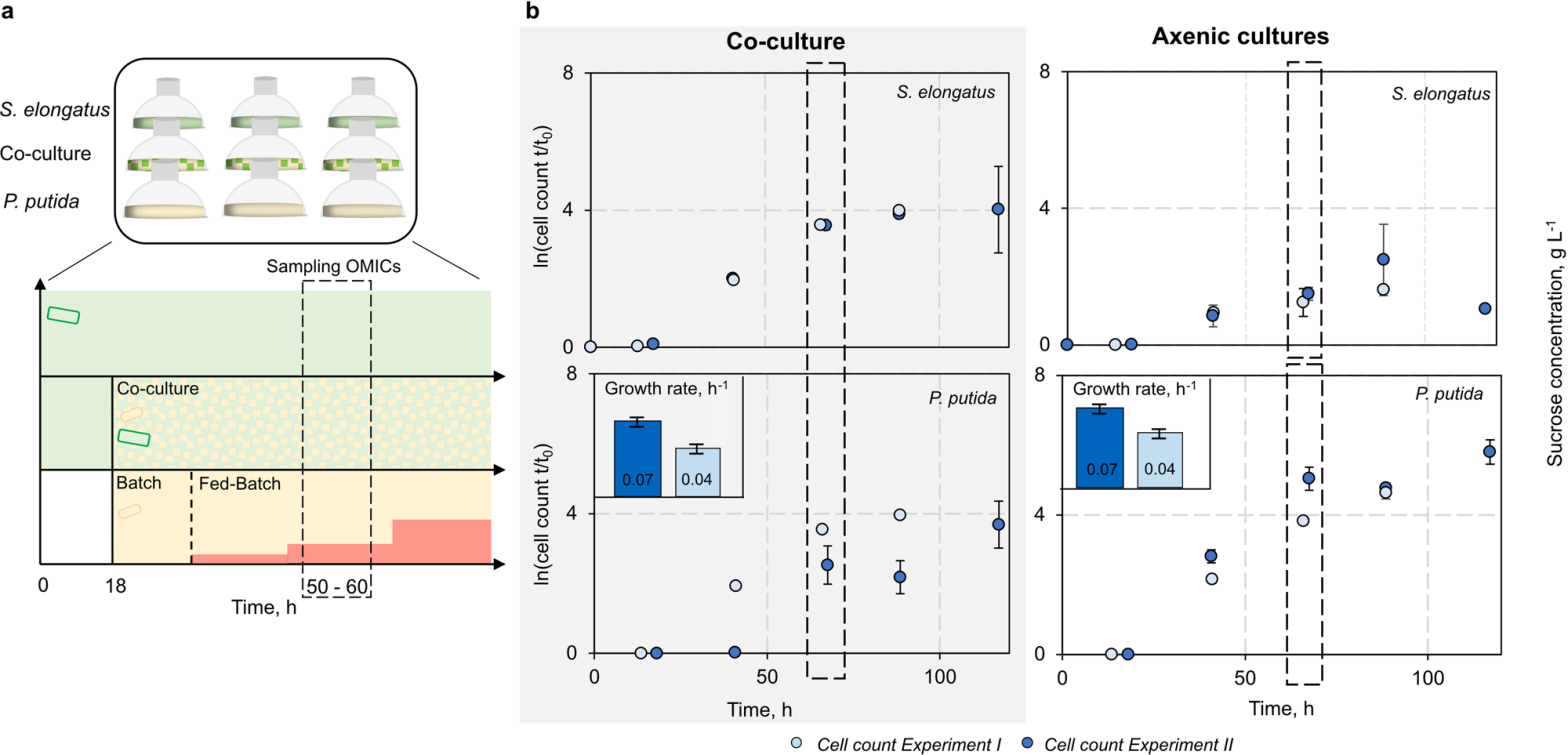
Reference experiment for the multi-OMICs analysis. **a** Schematic visualisation of the reference experiment consisting of three different settings, each performed in triplicates. The settings are: Axenic *S. elongatus cscB* culture (green), the co-culture (green and yellow), and *P. putida cscRABY* axenic culture (yellow). Red stages represent the feed with sucrose for axenically grown *P. putida cscRABY*. **b** Two reference experiments (Experiment I - light blue and Experiment II - dark blue) with the co-culture of *S. elongatus cscB* and *P. putida cscRABY* (shaded grey) and the respective axenic cultures. Depicted is the normed cell count; growth rates of *P. putida cscRABY* in the co-culture and the axenic culture are represented as small bar charts (calculated between 18–65 h). The dashed boxes indicate the sampling points for the multi-OMICs. *Experimental conditions*: 25–32 °C, 2% CO_2_, 95 mL BG11^+^ + 150 mM NaCl, volume 95 mL; exponential light profile: 120 µmol m^−2^ s^−2^ constant for 24 h followed by exponential rising with t_d_ *=* 52 h. Data is derived from *n* *=* 3 biologically independent experiments, and error bars represent the standard deviation (based on a sample) of the replicates, calculated using the *n* *−* 1 method.

To analyse the interplay between *P. putida cscRABY* and *S. elongatus cscB* and to find a hint at what might be the origin of the remarkable increase in the growth rate of *S. elongatus cscB* grown in co-culture, we aimed to compare the co-culture with the axenic cultures of *S. elongatus cscB* and *P. putida cscRABY*, respectively. Therefore, we set up a reference procedure in the 9-fold parallel photobioreactor system, which allowed us to have comparable conditions in all three different setups, each in biological triplicates, for the analysis of the transcriptome, proteome, and metabolome (Fig. 3a).

To achieve comparable growth rates of *P. putida cscRABY* in axenic cultures and in the co-culture, we adjusted an external sucrose feed for axenic *P. putida cscRABY* cultivations that mimicked the cyanobacterial sucrose secretion. In order to calculate the sucrose feeding rate, the biomass formation was connected with the sucrose uptake by the heterotrophic partner (calculations see Supplementary Note S5). However, achieving consistency was challenging as the growth rate of *P. putida cscRABY* in the co-culture showed a high variance between different experiments, assumingly due to variations in the temperature. Therefore, for the OMICs, two experiments that showed comparable temperature profiles (see Supplementary Note S6 and Supplementary Fig. S4) have been chosen, from now on referred to as EI and EII (Fig. 3b). Samples for transcriptomics and proteomics were derived from EII and samples for metabolomics from EI at 27.0 °C or 27.4 °C, respectively. Thus, for each of the OMICs experiments, external conditions, such as light or temperature, were identical, as all samples were taken from the same experiment conducted in the 9-fold parallel photo-bioreactor. Growth of the phototrophic partner was highly reproducible in the co-culture compared to the axenic culture (Fig. 3b, Supplementary Note S7 and Supplementary Fig. S5) in both experiments. As observed before, the growth was enhanced in the co-cultures compared to the axenic cultures. An early entry of the cyanobacterial cells into the stationary phase and visible photobleaching after 85 h was prevented. Furthermore, cells of *S. elongatus cscB* grown in co-culture had a smaller size, which fits well with the more than doubled specific growth rate (Table S2 and Supplementary Note S8).

The growth of *P. putida cscRABY* depends on the cyanobacterial sucrose secretion in the co-culture and on the sucrose feed in the axenic culture. Although the growth rates of *P. putida cscRABY* were different between both experiments, they did not differ within the same experiment (Supplementary Table S2). This was important for the -OMICs, as these were the conditions to be compared. No differences in the cell size of *P. putida cscRABY* in co-cultures or axenic cultures could be observed, which aligns with the expected outcome due to the same growth rate (Supplementary Fig. S6). We assumed sucrose to be the growth limiting factor in both cultures, and in fact, in the axenic cultures of *P. putida cscRABY* and the co-culture, no sucrose could be detected at the time point when the samples for the OMICs were taken (Supplementary Fig. S7).

As *P. putida cscRABY* is limited by the carbon source in all cultures, we set out to analyse the medium components citrate and phosphate and other commonly known overflow metabolites of *P. putida*. We detected a transient accumulation of acetate and ethanol, but both substances were completely taken up again at the end of the process, assumingly by *P. putida cscRABY* itself, as *S. elongatus cscB* does not harbour typical genes for acetate uptake. The BG11^+^ medium contains citric acid, which was completely consumed within 13.5 hours in all cultures. However, after 40 h, citric acid concentration increased again, particularly in cultures with *P. putida cscRABY*. At first glance, this is counterintuitive, as under carbon limitation, cells should coordinate their energy and maintenance needs. However, a recent study showed that *E. coli* secrets overflow metabolites to address carbon-nutrient imbalances^22^. Thus, apart from resulting from cell lysis, this might also be the case here (Supplementary Fig. S7).

A gross estimation of the photosynthetic activity of *S. elongatus cscB* is possible, as the amount of carbon fixed can be approximated by taking into account biomass production and sucrose accumulation (Supplementary Note S10). It has already been described by others that inducing sucrose secretion resulted in increased overall CO_2_ fixation in *S. elongatus cscB*^10,18,23^. This is explained by the idea that heterologously implemented sucrose production serves as a sink for the carbon captured in the Calvin cycle, thereby alleviating the so-called photosynthetic sink limitation. Sink limitation describes the situation when photosynthetic activity is reduced due to insufficient withdrawal of products from the Calvin cycle^17^. In the co-culture, the constant pull on the sucrose production by the heterotroph seems to lead to a more efficient utilisation of the captured carbon and thereby contributes to overcoming sink limitation. We observed that in the co-culture, the photosynthetic activity of *S. elongatus cscB* was even higher than under inducing conditions, as the growth rate surpassed that of the cells not expressing *cscB*, and as additionally heterotrophic growth was supported by sucrose secretion (Supplementary Table S3 and Supplementary Fig. S8).

### Overview of multi-OMICs in the co-culture process

**Fig. 4:**
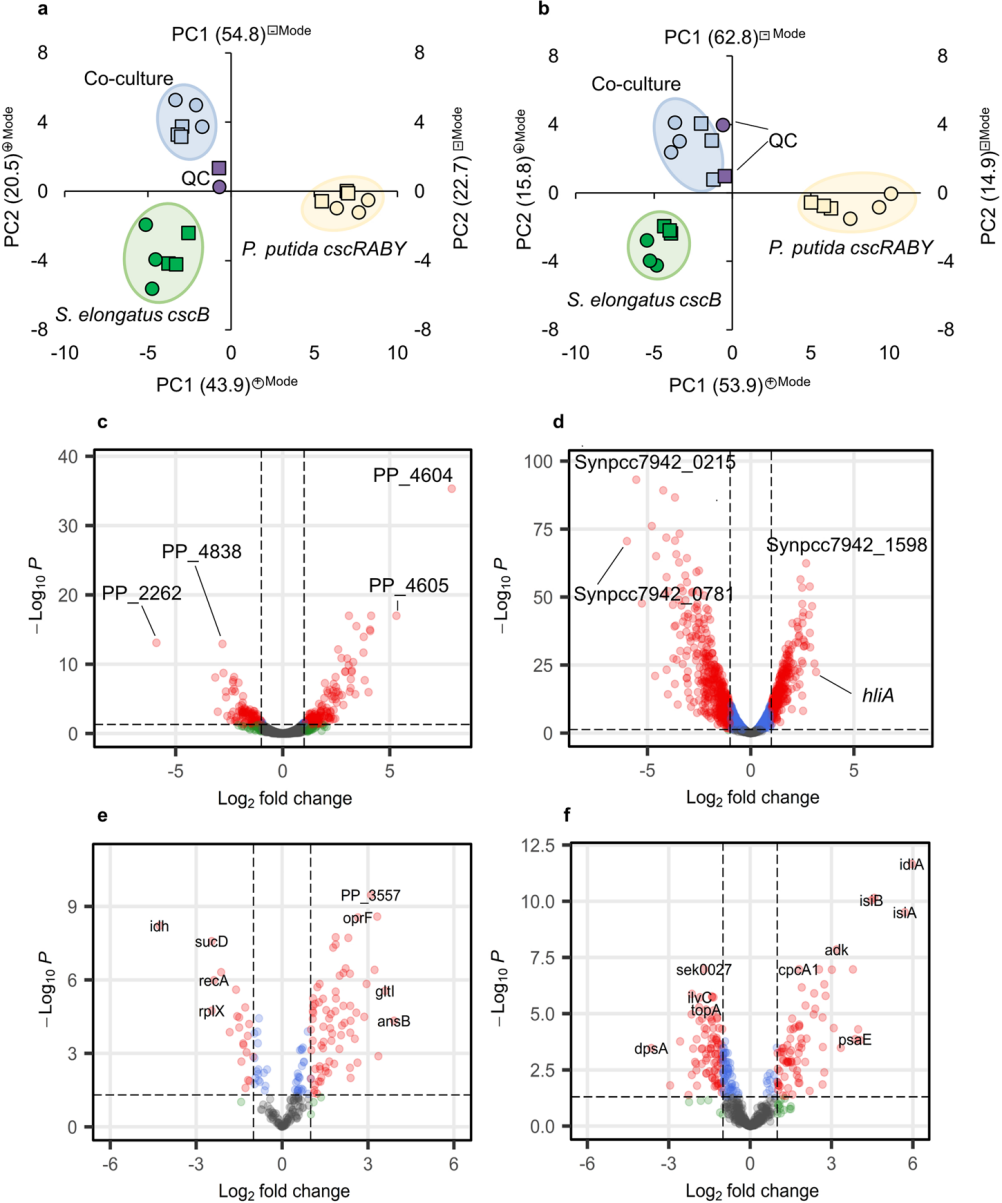
Principal component analysis (PCA) of metabolites. **a** RP-MS and **b** HILIC-MS measurements. The positive mode is depicted in circles, and the negative mode is depicted in squares. Green = *S. elongatus cscB*, yellow = *P. putida cscRABY*, blue = co-culture, purple = controls. **c**, **d** Volcano plots of the transcriptomics. **e**, **f** Volcano plots of the proteomics. In **c** and **e**, the co-culture is compared to the axenically grown *P. putida cscRABY*; in **d** and **f**, the co-culture is compared to the axenically grown *S. elongatus cscB*. Transcripts and proteins in red circles meet the threshold for both log_2_-FC and *p*-value. Transcripts and proteins depicted in blue fulfil the threshold only for the *p*-value, while transcripts or proteins in green meet the threshold only for log_2_-FC. Grey represents transcripts and proteins that do not meet both thresholds. The thresholds are indicated in all plots by dashed lines.

As described above, the presence of *P. putida cscRABY* in the co-culture had a positive effect on the growth of *S. elongatus cscB*. In the next step, we aimed to get insights into the inter-species interaction in the co-culture. This is not only interesting from a fundamental research perspective but will also contribute to enhancing our understanding of co-culture stability and, eventually, even help to improve the production of value-added products in co-cultures in general. Therefore, transcriptomics, proteomics, and metabolomics were performed (see Supplementary Note S11). Samples for multi-OMICs were taken at ~60 h and processed as described in the methods section. The time point was chosen to be in the second half of the growth phase before cells entered the stationary phase to ensure a sufficiently high number of cells for the analysis (compare Fig. 3a). The co-culture was considered as the case of interest and compared to the axenic cultures, which were considered as the controls. This provided us with a snapshot to describe the cellular status at the time of sampling. In all the datasets, we could pinpoint distinct clusters that were specific for either the co-culture or the axenic cultures, respectively. Exemplarily, this is demonstrated in Fig. 4a, b for the metabolome data, which was used for a principal component analysis. Thresholds for differently expressed genes (DEGs) and differently abundant proteins (DAPs) were set at |log_2_(FC)| >1.0 and a *p*-value (adjusted, false discovery rate (FDR) corrected) <0.05. For different abundant metabolites, the threshold was the adjusted *p*-value of 0.05 and a mean difference of 0.3.

Comparing the transcriptome of the co-culture to the axenic culture, in *P. putida cscRABY*, a total of 488 differently expressed genes (DEGs) were identified, of which 303 were up-regulated, and 145 were down-regulated (Fig. 4c). In *S. elongatus cscB* a total of 790 DEGs were identified. Of these 324 genes were found to be up-regulated, while 466 were down-regulated in comparison to axenically grown cells (Fig. 4d). Gene set enrichment analysis based on the KEGG Orthology database indicated that in *P. putida cscRABY*, pathways involved in arginine biosynthesis, carbon metabolism, glyoxylate and dicarboxylate metabolism, as well as two-component systems, were impacted by the presence of the co-culture partner (Supplementary Fig. S9). For *S. elongatus cscB*, pathways involved in arginine/proline metabolism, photosynthesis, pyruvate, glycolipid, and biosynthesis of secondary metabolites were identified in the analysis to be the most affected ones.

The analysis of the proteome revealed 69 proteins in *P. putida cscRABY* to be more abundant, and 27 proteins showed less abundance when compared to the axenic culture (Fig. 4e). In *S. elongatus cscB*, a total of 92 proteins were identified to be more abundant in co-culture, and 91 proteins were less abundant (Fig. 4f). The majority of proteins identified in *P. putida cscRABY* belong to the category amino acid metabolism and transport, and the majority identified in *S. elongatus cscB* belong to the group of photosynthesis or the category stress. This trend was also observed in the transcriptome (see above). In general, the match between transcriptomics and proteomics regarding the identification of processes and direction of regulation is the range of what is described as the regular magnitude^24^ (Supplementary Fig. S10).

Analysing the metabolome, in total, 876 features could be identified in the cells with the HILIC-MS measurements (− and + MS-mode), and 1013 features were identified with RP-MS (− & + MS-mode). When comparing the features obtained in the co-culture grown cells to those identified *P. putida cscRABY* grown in axenic culture, 336 features for HILIC (− & + MS-mode) and 427 for RP (− & + MS-mode) fulfilled the conditions set (see Supplementary Fig. S11 for Volcano plots). A comparison of the features identified in the co-culture grown cells to those obtained in *S. elongatus cscB* grown in axenic culture revealed 143 features that fulfilled the threshold set for HILIC-MS measurement (− & + MS-mode) and 254, which fulfilled it for the RP-MS (− & + MS-mode). Most features were more abundant in the co-culture than in the respective axenic culture. By reference measurements, some metabolites could be identified (Supplementary Fig. S12). Most of them participate in sugar metabolism or belong to the group of phospholipids, amino acids, or fatty acids.

### Cellular processes affected by the co-culture partner: Core metabolism and photosynthesis

**Fig. 5:**
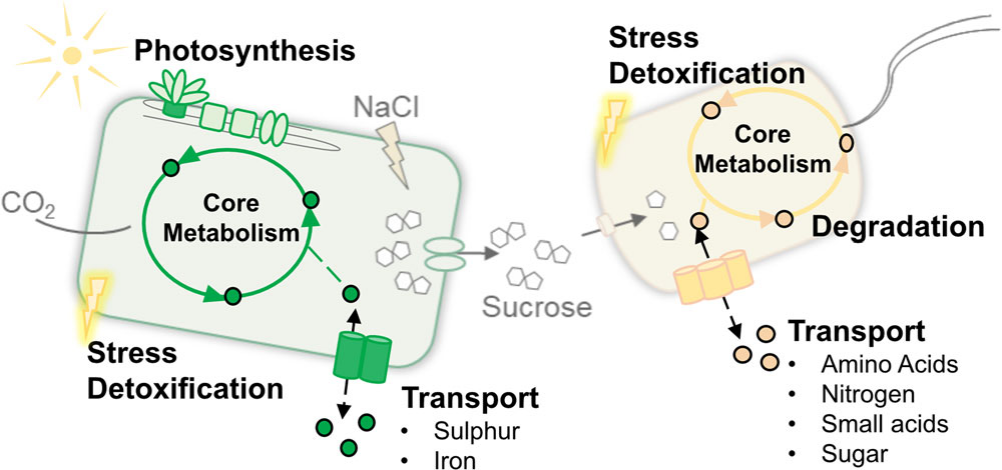
Overview of the cellular processes affected by the co-cultivation. In *S. elongatus cscB* (green), the major impact of the presence of the heterotrophic partner was found in the core metabolism and photosynthesis. Furthermore, stress or detoxification-related processes and the transport of sulphur and iron were also affected. In *P. putida cscRABY* (yellow), processes in the core metabolism, transport of amino acids, nitrogen, small acids, and sugars, as well as in stress, detoxification and degradation were affected by the presence of the phototrophic partner.

The analysis of the multi-OMICs data yielded a large number of genes and proteins that were differentially expressed or abundant when comparing co-cultivated cells to those cultivated in axenic cultures. Additionally, on the metabolome level, some differences were identified. To get a first overview of the cellular processes that were mainly affected by the presence of the respective co-culture partner, we sorted the DEGs, DAPs, and metabolites into different groups according to their putative function (Fig. 5). In *P. putida cscRABY* the presence of the phototrophic partner led to changes in various cellular processes, namely in the core metabolism, transport of amino acids, nitrogen, small acids, and sugars, but also in the general stress response, detoxification, and degradation. In *S. elongatus cscB*, we found that the presence of the heterotrophic partner likewise had an effect on the core metabolism but also on photosynthesis, which was somehow expected as *S. elongatus cscB* exhibited a higher growth rate in the co-culture. Furthermore, other processes connected to stress, detoxification, or transport of sulphur and iron were affected. In the following, these processes will be discussed in more detail.

**Fig. 6:**
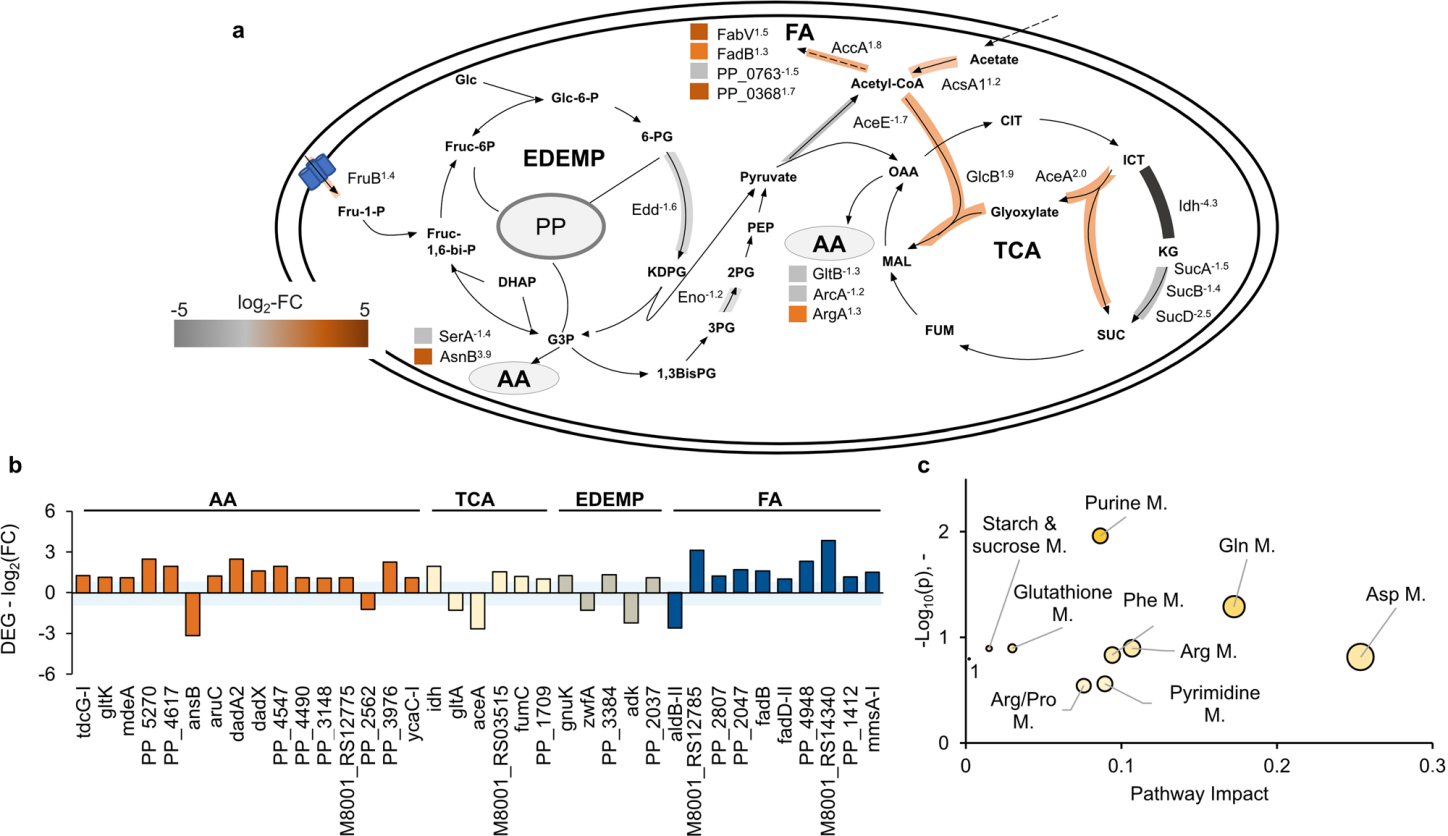
Changes in the central carbon metabolism of *P. putida cscRABY* grown in co-culture. **a** Proteome level: Differentially abundant proteins (DAPs) are highlighted in orange (more abundant) or grey (less abundant). Superscript numbers indicate the log_2_-fold change (log_2_-FC). **b** Transcriptome level: DEGs encoding proteins putatively involved in AA (amino acid) synthesis and degradation, or the TCA (tricarboxylic acid) cycle, the EDEMP (Entner-Doudoroff-Embden-Meyerhof-Parnas)-cycle, or the FA (fatty acid) synthesis and degradation. The blue shaded area indicates the p-value of 0.05. **c** Metabolome level: metabolites identified by reference measurements comparing the co-culture to *P. putida cscRABY* and grouped into pathways by a pathway enrichment analysis. Data was obtained by using the metabolic pathway analysis of MetaboAnalyst 5.0. Abbreviations: 1 = Phe/Tyr Metabolism, amino acids are abbreviated with the three-letter code, and M stands for metabolism.

In *P. putida cscRABY* several genes, proteins, and metabolites with a potential function in the core metabolism were identified to be affected by the presence of *S. elongatus cscB* in the co-cultivation. More specifically, on the proteome as well as on the transcriptome level, processes that are connected to the amino acid (AA) synthesis or degradation, the TCA cycle, or to the fatty acid (FA) metabolism were affected (Fig. 6a, b). Additionally, some genes encoding proteins involved in the Entner-Doudoroff-Embden-Meyerhof-Parnas (EDEMP) cycle were differentially regulated (Fig. 6b). On the metabolome level, the metabolites identified mainly belonged to the group of amino acids (Fig. 6c). As a rhizobacterium, *P. putida* is specialised for the uptake and metabolisation of amino acids^25^. In line with this, it was not surprising that the expression of genes, as well as the abundance of proteins connected to amino acid metabolism, was affected by the presence of the co-culture partner. On the proteome level, the asparagine synthetase AsnB, responsible for the conversion of aspartic acid into asparagine, showed the biggest differences with a log_2_-FC 3.9 (Fig. 6a). The corresponding transcript *asnB*, however, was down-regulated (Fig. 6b, PP_2453 log_2_-FC -3.1). This demonstrates a prevailing issue of multi-OMICs analysis, which is that the correlations between proteomics and transcriptomics are only moderate^24^. However, these differences may also indicate a post-transcriptional control. On the metabolome level, the amino acids L-phenylalanine, L-glutamic acid, L-aspartic acid and L-glutamine were identified to be more abundant in the co-culture compared to *P. putida cscRABY* grown axenically (Fig. 6c and Supplementary Fig. S12). However, the metabolites were identified in all cells grown in co-culture and, therefore, cannot be assigned specifically to one of the co-culture partners. Another sector of the core metabolism that was affected is the TCA cycle. On the proteome level, lower protein abundances of Idh and SucA/B/D went together with a higher abundance of the proteins AceA and GlcB, hinting towards a shut-down of the TCA cycle and a redirection of the metabolic flux through the glyoxylate cycle (Fig. 6a). This is known to happen in *P. putida* when degradation of aromatics or xenobiotics is necessary ^26^. In the transcriptome, the contrary is observed with a slight up-regulation of *Idh* (PP_4012 log_2_-FC 1.9) combined with the down-regulation of the transcript encoding AceA (PP_4116 log_2_-FC -2.64).

Taken together, the core metabolism of *P. putida cscRABY* is influenced by the presence of the co-culture partner, particularly affecting processes belonging to the amino acid metabolism and the TCA cycle. In general, the central metabolism can reflect different metabolic states of the cell, as it was described for cells growing on mixtures of carbon sources^26^. Differences in the environmental conditions, brought about by cultivation in the co-culture, might, therefore, lead to changes in the core carbon metabolism or in its periphery, such as the amino acid or fatty acid metabolism. Alterations in the metabolism were further corroborated by the identification of secretion and re-uptake of some metabolites, such as transient accumulation of ethanol, acetate, and citrate in the supernatant during different cultivation phases (Supplementary Note S9).

**Fig. 7:**
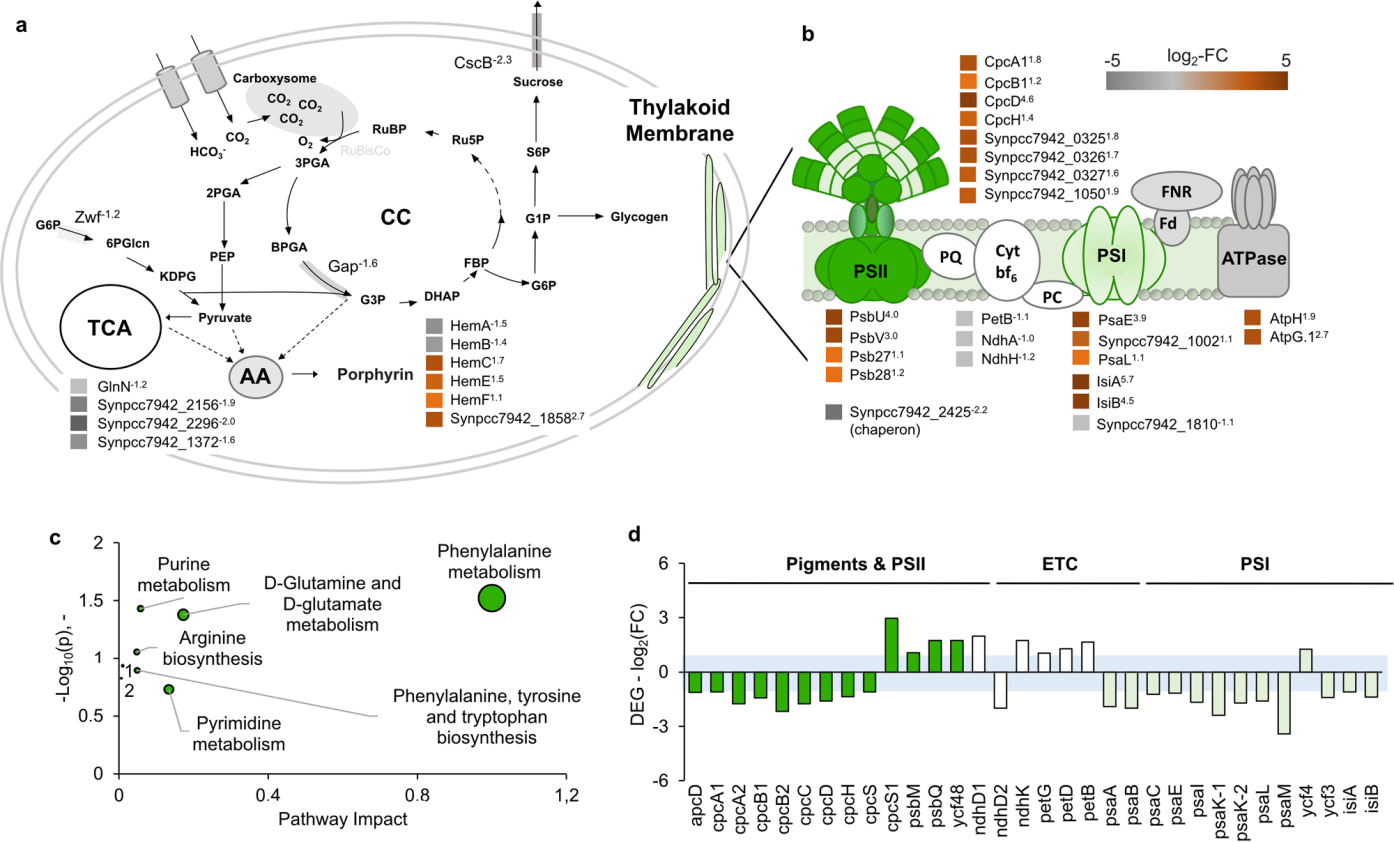
Changes in the central carbon metabolism and photosynthesis of *S. elongatus cscB*. **a** Proteome level: Schematic cell of *S. elongatus cscB* with changes in the amino acid (AA) metabolism, porphyrin metabolism, (CC) Calvin cycle, and in photosynthesis (TCA = tricarboxylic acid cycle). DAPs are shown in orange (more abundant) or grey (less abundant). Superscript numbers indicate the log_2_-fold change (log_2_-FC). **b** Changes in the photosynthesis apparatus on the proteome level: PSII = photosystem II, PQ = plastoquinone, Cyt-bf_6_ = cytochrome bf_6_, PC = Plastocyanin, PSI photosystem I, FNR = ferrodoxin-NADP^+^ reductase, and Fd = ferrodoxin/flavodoxin. DAPs are shown in orange (more abundant) or grey (less abundant). Superscript numbers indicate the log_2_-fold change (log_2_-FC). **c** Metabolome level: Data were obtained by using the metabolic pathway analysis of MetaboAnalyst 5.0. Abbreviations: 1 = Glutathione metabolism, 2 = Glyoxylate and dicarboxylate metabolism. **d** Changes in the photosynthesis apparatus on the transcriptome level: DEGs are subdivided into the groups pigments & PSII, ETC = electron transfer chain, and PSI. The blue shaded area indicates a *p*-value of 0.05.

When looking at the processes affected in *S. elongatus cscB* by the presence of *P. putida cscRABY*, it has to be kept in mind that the growth rates of axenically grown cells and cells grown in co-culture differ by a factor of about two. Variations in transcripts or proteins can be the consequence of the higher growth rate or arise from the interaction with the co-culture partner, which encompasses specific interactions as well as non-specific effects, such as shading or the response to metabolic signals, which might arise from secreted metabolites and/or consumed resources. These effects might also be entangled, as a positive interaction could lead to a higher growth rate. The higher growth rate of *S. elongatus cscB* in the co-culture is reflected by an up-regulation of many growth-associated genes, such as ribosomes, tRNAs, and polymerases, as it was observed at the transcriptional level (Supplementary Note S12 and Supplementary Fig. S13). This was not the case for *P. putida cscRABY*, showing that a similar growth rate in axenic culture and co-culture leads to a similar expression pattern of these genes. The higher abundance of amino acids in the metabolome of the co-culture, which was already mentioned above, could also be the consequence of the increased metabolic activity of the cyanobacterium. However, attributing core metabolites to a specific co-culture partner is not possible. Analysing the core metabolism of *S. elongatus cscB* in more detail, only a few proteins showed different abundance. They can be grouped into proteins being involved in the porphyrin metabolism, the Calvin cycle, or photosynthesis (Fig. 7a). Interestingly, the heterologously expressed sucrose transport protein CscB was less abundant in *S. elongatus cscB* when grown in co-culture. This is on the first glace counterintuitive, as its transcription is regulated by the IPTG inducible *P*_*lacUV5*_ promoter and should, therefore, be constant. No information on its transcript level is available, as the *cscB* gene was not included in the transcriptomic analysis. We explain the lower abundance of the CscB protein by the higher growth rate of *S. elongatus cscB* in the co-culture. Assuming the total amount of the CscB protein produced remains constant, but cells divide more rapidly, the CscB protein is distributed across a larger number of cells, which results in a lower abundance. In the metabolome of the co-culture grown cells, we detected a higher amount of disaccharide, which could be sucrose, which might hint towards a higher accumulation of sucrose in the cytoplasm of the cyanobacterial cells due to decreased export activity. Other metabolites identified in the metabolome of co-culture grown cells include compounds assigned to the biosynthesis of amino acids or purine and pyrimidine metabolism (Fig. 7c).

Looking at genes and proteins involved in photosynthesis, the effect of the different growth rates, when grown axenically or in co-culture, becomes even more obvious. In the proteome, the most pronounced change was the increased abundance of phycobiliproteins. Additionally, the pigment-proteins phycocyanin and allophycocyanin, which are present in the light-harvesting complex, were also more abundant (Fig. 7b). In general, the light harvesting complexes are connected to photosynthetic activity and growth. However, here, only a few proteins of the Photosystem I (PSI), Photosystem II (PSII), and the connecting electron chain consisting of the NAD(P)H-dehydrogenase-like complex (NDH) and Plastocyanin (PQ) were detected. In the transcriptome, the opposite effect was observed: Genes encoding proteins forming the PSI and PSII and the phycobiliproteins were down-regulated, whereby genes coding for the connecting NDH/PQ complex were up-regulated (Fig. 7d). A likely explanation for the difference observed is provided again by the different growth rates observed in the two culture conditions. The axenically grown cells of *S. elongatus cscB* displayed reduced growth and manifested phenotypically evident stress effects. At the time point of sampling, cells grew linearly, suggesting a non-constant growth rate and, consequently, a dynamic state of cellular processes, whereas the cells grown in co-culture exhibited exponential growth with a constant growth rate, indicating an intracellular steady state of transcripts, proteins, and metabolites. As a result, transcripts and proteins may be differently affected when comparing the co-culture to the axenic cultures of *S. elongatus cscB*. Photosynthesis is highly regulated, for instance, by the PSI:PII ratio^27^. In order to cope with excess energy, photosynthetic organisms regulate their electron transport chain (ETC) to prevent the production of ROS. Another mechanism for encountering photooxidative stress in high-light conditions involves the protein pair IsiA and IsiB. Both proteins were more abundant in the co-culture, IsiA with a log_2_-FC of 5.7 and IsiB with a log_2_-FC of 4.5, which was amongst the highest increases detected at the protein level (Fig. 7b). IsiA is annotated as an iron stress induced chlorophyll-binding protein and IsiB as a flavodoxin. Consistent with iron induced stress, two other proteins linked to iron limitation, IdiA and IrpA were notably more abundant in the co-culture grown cyanobacteria (see Table 2). However, we could not detect a stronger iron limitation for *S. elongatus cscB* in the co-culture, as iron supplementation or limitation had no discernible effect on the cultures (Supplementary Note S13 and Supplementary Fig. S14), nor was the higher protein abundance of IsiA and IsiB reflected in the transcriptome. Additionally, at the proteome level, some proteins associated with porphyrin biosynthesis were identified, with the majority showing higher abundance (Fig. 7a), potentially linked to observed variations in photosynthesis.

### Cellular processes affected by the co-culture partner: Transport

**Fig. 8:**
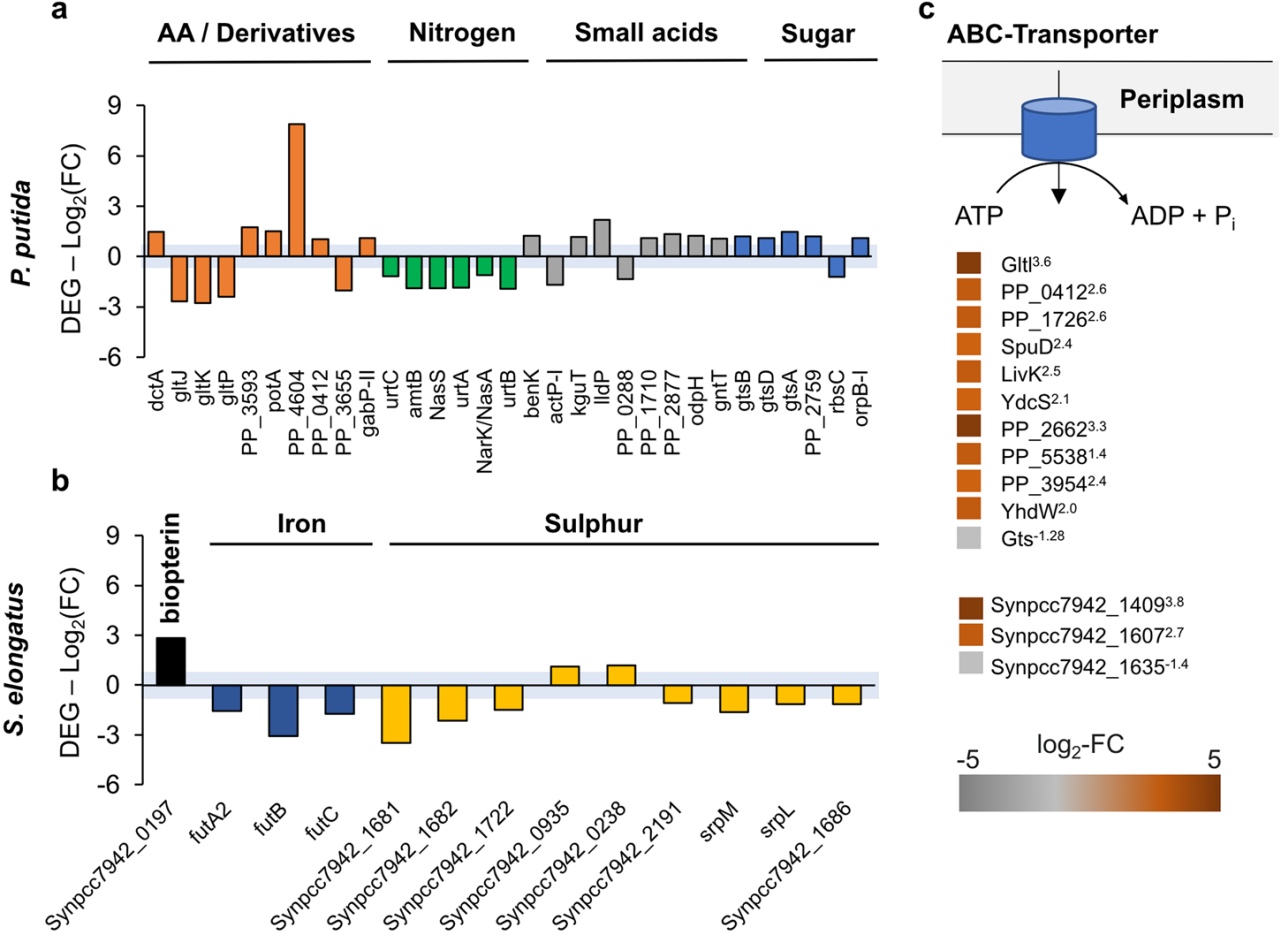
Differential expression of transporters in the co-culture. **a** DEGs in *P. putida cscRABY* grouped according to the transported substrate: AA (amino acids), nitrogen, small acids, and sugars. The blue area indicates a *p*-value of 0.05. **b** DEGs in *S. elongatus cscB* classified into two major groups: iron and sulphur transport. The blue area indicates a *p*-value of 0.05. **c** Proteome level: DAPs in *P. putida cscRABY* (above) and *S. elongatus cscB* (below) connected to transport. The colour bar indicates the changes in the protein abundance. Superscript numbers indicate the log_2_-fold change (log_2_-FC).

Microbes often rely on their capacity to efficiently utilise a wide range of resources, which can be a critical factor in their competitiveness and overall performance in relation to other microorganisms. Consequently, they have developed numerous strategies for acquiring compounds from their surrounding environment. Most microbial interactions require a form of uptake of substrates, signals in diverse forms, or toxins. In line with this, we have identified various transporters to be differentially regulated in the co-culture versus axenic cultures (Fig. 8). In general, we observed that in *P. putida cscRABY*, transporters are more likely up-regulated, whereas the opposite is the case in *S. elongatus cscB*. This might originate in the individual lifestyles of each of the co-culture partners, as a strict autotrophic and anabolic mode needs less transport of organic carbon compounds compared to a heterotrophic lifestyle.

In *P. putida cscRABY* at the transcriptome level, the DEGs related to transport are diverse and include genes encoding putative transporters for amino acids, nitrogen, small acids, and sugars (Fig. 8a). While in the group of amino acid transporters, the transcription of the corresponding genes showed regulation in both directions, in the group of nitrogen transport transcription was down-regulated and in the groups of small acids and sugar transport, transcription was mainly induced. The most highly up-regulated transcript was PP_4604, found in the group of amino acid transport. It encodes a putative transporter belonging to the EamA family, which in *E. coli* is related to transport of cysteine-derivatives^28^. Directly downstream of this gene, a gene encoding an AraC-type regulator (PP_4605, log_2_-FC 5.3), was found to be highly up-regulated as well. A putative connection between these two genes is predicted by the string-database. Down-regulation was observed for some genes encoding ABC-transporters for glutamate/aspartate uptake (*gltJ* log_2_-FC -2.6, *gltP* log_2_-FC −2.4, and *gltK* log_2_-FC −2.7), whereas genes encoding proteins connected to the transport of other amino acids derivatives, such as putrescine or spermidine (*potA* ATB-binding and PP_0412 substrate binding with a log_2_-FC of 1.5 and 1.0) were up-regulated. At the proteome level, the latter one, PP_0412, was also identified to be more abundant. Additionally, the proteins SpuD and YhdW, annotated as polyamide transporter, were found to be more abundant in *P. putida cscRABY* (Fig. 8c). The down-regulation of the transcription of genes encoding nitrogen and urea transporter, for instance *amtB* encoding an ammonium transporter, or *urtABC*, coding for a urea transporter, fit well to the downregulation of the *ureABCD* cluster encoding a urease for urea degradation. In line with this, at the protein level, the global regulators NtrB and NtrC, which are responsible for nitrogen regulation, are less abundant.

At first glance, it is not intuitive, that many genes involved in transport are differentially regulated in the co-culture, as, neglecting the small amount of citrate in BG11^+^ medium, the sole carbon source is sucrose secreted by the phototrophic partner. However, at a global ecological scale, cyanobacteria including *Synechococcus spp*., are well-known to drive marine bacterial communities because they are the main suppliers of organic matter due to cell death, cell lysis and leakiness to photosynthate or exudates^16,29^. In artificial seawater medium (nutrient rich) *Synechococcus* cultures accumulated up to 200 µg mL^−1^ carbohydrates and 400 µg mL^−1^ proteins^16^. Furthermore, it is described that cyanobacteria can secret amino acids and other components. For example, in the supernatant of *S. elongatus* CCMP 1631 tryptophan and phenylalanine were found^5,30^. Moreover, *P. putida* is able to colonise plant roots and was shown to exhibit advanced chemotaxis towards polyamides, which are a component of complex root extrudates^31,32^. Thus, in a more general view, it is plausible that transporters for carbon, carbon-nitrogen compounds, or nitrogen in *P. putida cscRABY* are affected by the presence of the phototrophic co-culture partner.

In *S. elongatus cscB* the DEGs encoding proteins related to transport mainly belong to the group of transporters for iron or sulphur (Fig. 8b). Almost all of them were down-regulated, with the exception of Synpcc7942_0197, which was the most highly up-regulated gene, encoding a putative folate/biopterin family MFS transporter (log_2_-FC 2.8). Pterins are ubiquitously occurring molecules, which are needed by cyanobacteria for pigment generation, phototaxis, and UV protection^33^. In the group of genes related to iron transport, the *futABC* operon encoding siderophores responsible for iron uptake (*futA2* log_2_-FC −1.6, *futB*, log_2_-FC −3.1, and *futC* log_2_-FC −1.74) was down-regulated (Fig. 8b). In the group of genes encoding sulphur transporters, the strongest down-regulation was observed for Synpcc7942_1681, annotated to encode a sulphate/sulfonate transporter. Synpcc7942_1682, and Synpcc7942_1722, also encoding putative sulphate/sulfonate transporters, were likewise down-regulated. Other genes encoding putative sulphite exporters were slightly up-regulated (Synpcc7942_0935, Synpcc7942_0238). Sulphur is an essential element for microbes and participates in iron-sulphur clusters, a common co-factor of proteins, in many important physiological processes including photosynthesis, DNA/RNA modification, and purine metabolism^34^. Sulphite is cell toxic and arises from the intracellular breakdown of metabolic products, including sulphur-containing amino acids, which boosts ROS generation^35^. Regulation of the transcription of genes potentially involved in sulphur or sulphite transport hints towards differences in the complex processes of sulphur homeostasis in *S. elongatus cscB*, when grown in co-culture with *P. putida cscRABY*. In conclusion, iron and sulphur transport seems to be down-regulated in *S. elongatus cscB* when growing together with the heterotrophic partner.

On the protein level, only three proteins associated with transport were identified to be differentially abundant. Two of them, annotated as substrate-binding protein of an iron transport system (Synpcc7942_1409) and as a hypothetical porin (major outer membrane protein, Synpcc7942_1607) showed a higher abundance whereas another porin (Synpccc7942_1635) was less abundant in the co-culture grown *S. elongatus cscB* cells (Fig. 8c).

### Cellular processes affected by the co-culture partner: Detoxification, degradation and stress

Next, we analysed the group of regulated genes and proteins, that can be functionally related to detoxification, degradation, and stress. Hays et al. had observed a negative effect of *S. elongatus cscB* on the growth of respective heterotrophic partner^11^, however as described above, we have not seen this effect on *P. putida cscRABY* in small-scale experiments. In the co-culture setup, both organisms experience multiple situations that could cause different types of stress. One situation they have to cope with is the increased salinity conferring high ionic strength and external osmotic pressure. Additionally, light can induce oxidative stresses and the adaptation to changes in the illumination can be a further stress factor. However, these external factors are comparable in both conditions, the axenic cultures and the co-cultivation, thus differences that are identified in transcript or protein abundance related to stress signals are regarded to be specific for the presence of the respective co-culture partner.

**Fig. 9:**
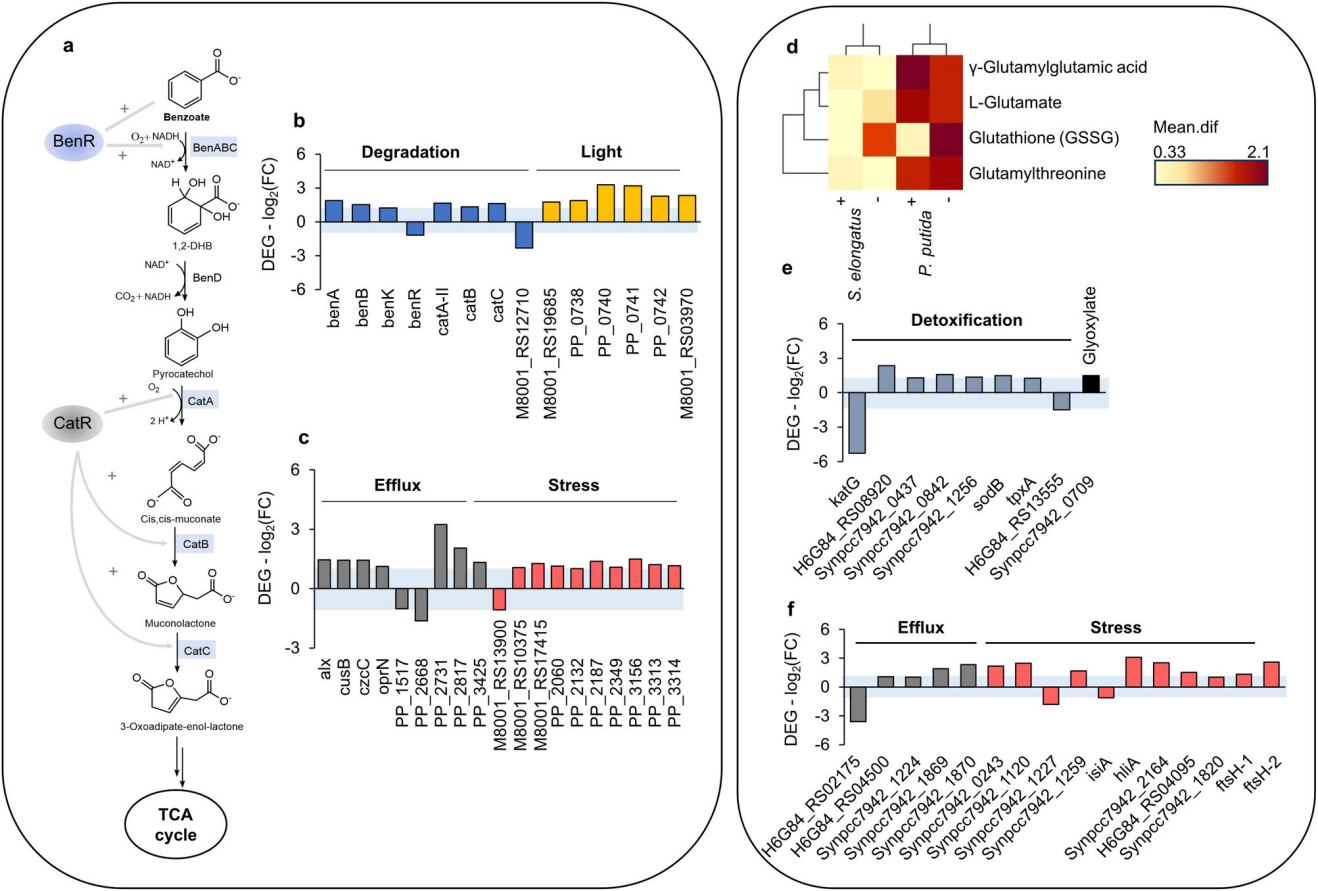
Effects on detoxification, degradation, and stress response. **a** Schematic visualisation of the degradation of benzoate and assimilation in the TCA (tricarboxylic acid) cycle by proteins encoded by the benABCD and catABC-operon in P. putida cscRABY cells grown in co-culture. Blue shading marks differential expression of the corresponding genes, and grey arrows indicate regulation by BenR and CatR. **b** DEGs annotated to be involved in aromatic degradation and light-induced stress in P. putida cscRABY grown in co-culture. **c** DEGs annotated to be involved in efflux (detoxification) or general stress in P. putida cscRABY grown in co-culture. **d** Heat map for metabolites measured with HILIC in positive (+) and negative (-) ionisation modes. Mean difference is presented in a colour code from red highest (2.1) to yellow lowest (0.33). Shown is the comparison of the metabolites identified in the co-culture to the respective axenic cultures of S. elongatus cscB or P. putida cscRABY. **e** DEGs annotated to be involved in ROS detoxification or glyoxylate degradation of S. elongatus cscB. **f** DEGs annotated to be connected to efflux and stress in S. elongatus cscB.

Our data indicate that several processes assumingly connected to stress in *P. putida cscRABY* are affected by the presence of *S. elongatus cscB* (Fig. 9). More specifically, processes involved in the degradation of compounds, in the stress response induced by light, in the efflux of (toxic) substances, or in the general stress response were impacted. A general trend towards up-regulation of transcription was observed. For instance, the transcription of the genes belonging to the *ben*- and *cat*-operons encoding enzymes responsible for the degradation of benzoate were up-regulated (Fig. 9a, b). The gene encoding the AraC-type regulator BenR, however, was slightly down-regulated (log_2_-FC −1.2).

The transcription of a gene cluster (PP_0738 to PP_0742) that might be connected to light-induced stress was found to be up-regulated (Fig. 9b): One of its genes (PP_0739) encodes a putative deoxyribodipyrimidine photolyase and another PP_0740 encodes a putative MerR family transcriptional regulator of light-inducible genes, known as PplR1^36^. As the illumination was identical for the axenic culture of *P. putida cscRABY* and the co-culture, the light intensity per cell assumingly was lower in the co-culture due to the higher cell densities. Therefore, changes in the expression of these genes might be traced back to a different stress situation caused by the presence of the co-culture partner.

Most of the genes that were differentially expressed and are associated with the efflux of substances are up-regulated (Fig. 9c). Many are annotated to encode putative resistance-nodulation-division (RND) efflux pumps, such as Mex-RND and TolC-RND, which are responsible for the removal of toxic compounds. The highest up-regulation of transcription was detected for the genes PP_2817 and PP_2731 encoding putative multidrug efflux pumps with a log_2_-FC of 2.1 or 3.2, respectively. Furthermore, genes encoding proteins that can be connected to perceiving and combatting stress were differentially regulated, most of them showed upregulation in the co-culture. As mentioned above, ROS derived from the cyanobacterium’s photosynthesis is likely to be one of the major stress factors for heterotrophic partners. In line, one gene encoding a catalase (PP_2887 log_2_-FC 1.2) was found to be slightly up-regulated and, on the protein level, the catalase KatG was more abundant. However, only one of the two major cellular ROS degrading regulators, SoxR (PP_2060 log_2_-FC 1.1), was found to be marginally up-regulated. Another mechanism in the antioxidant defence is the glutathione metabolism^11,37^. However, no genes encoding proteins associated with glutathione metabolism could be identified to be differentially regulated, though, on the metabolome level, metabolites belonging to the glutathione metabolism were detected in the co-culture cells (Fig. 9e). The transcription of genes belonging to the *cop* and *czc*-operons was mainly up-regulated (Supplementary Note S14 and Supplementary Table S4). Their gene products are involved in copper homeostasis and the cytoplasmic detoxification of copper and silver ions, a vital process controlled by the CopR/CopS two-component system (Supplementary Fig. S15). Taken together, these findings indicate that *P. putida cscRABY* experienced a general stress situation, which is also corroborated by the up-regulation of genes encoding putative transcriptional regulators connected to stress^38^ (e.g. PP_0740 log_2_-FC of 3.3). We assume that the heterotrophic partner still has some capacity left to react to stresses, as certain stress answers, for example the glutathione metabolism, do not yet seem to be affected by the co-cultivation. This underlines that *P. putida cscRABY* is a well-fitting co-culture partner for *S. elongatus cscB* due to its natural tolerance towards all different kind of stresses.

By analysing the genes, proteins, and metabolites related to the stress response in *S. elongatus cscB* we have identified processes involved in redox reactions, efflux, general stress and ion homeostasis (Supplementary Note S15 and Supplementary Table S5). As already mentioned above, one of the key compounds to combat redox stress is glutathione, and oxidised glutathione (GSSG), L-glutamate and γ-glutamylglutamic acid and these were more abundant in the co-culture cells compared to either axenic culture (Fig. 9d). the transcription of genes encoding putative glutathione peroxidases (Synpcc7942_0437 and H6G84_RS08920) or a thioredoxin peroxidase *tpxA* (log_2_-FC 1.3) were up-regulated in cyanobacterial cells grown in the co-culture (Fig. 9e). Another way to handle oxidative stress is by the Glutathione-independent degradation of H_2_O_2_, performed enzymatically by catalases, peroxidases, and superoxide dismutase. Interestingly, the transcript of the catalase KatG was considerably down-regulated with a log_2_-FC -5.36 in the co-culture growing cyanobacterium. However, the superoxide dismutase SodB was up-regulated with a log_2_-FC 1.5 in *S. elongatus cscB* grown in co-culture (Fig. 9f).

As already observed for the heterotrophic partner, alterations were also found in the sector of efflux processes and general stress (Fig. 9f). DEGs encoding different types of efflux transporters, such as HlyD-family efflux transporters (Synpcc7942_1224) or RND efflux transporters (Synpcc7942_1869, Synpcc7942_1870) were mostly up-regulated in the co-culture, as was the transcription of genes that encode proteins putatively involved in stress response. In general, in cyanobacteria, high-light-inducible proteins (Hlip) are expressed in response to various exogenous stresses, including already moderate light intensity^39,40^. In this study, two transcripts encoding these proteins were also found to be up-regulated (Synpcc7942_1997 and Synpcc7942_1120) (Fig. 9f). Three FtsH proteases, responsible for protein homeostasis of the thylakoid membrane in photooxidative stress situations, were up-regulated on the transcriptional level (Synpcc7942_1820, Synpcc7942_0998, and Synpcc7942_0942), but on the protein level the proteins FtsH, FtsH.1, and FtsH.3 (Synpcc7942_0297, Synpcc7942_0942, and Synpcc7942_0998) were less abundant in the co-culture (Fig. 9f for transcripts and Supporting Information S15 for proteins). This is another example of the discrepancies between transcriptomics and proteomics results^24^.

Taken together, both co-culture partners showed differential regulation in processes connected to various stresses, detoxification and degradation when grown together. Even though *S. elongatus cscB* suffered from severe photobleaching and reduced growth in the axenic culture, the transcription of many genes encoding proteins involved in stress response was mostly up-regulated in the co-culture and not vice versa.

In summary, the multi-OMICs analysis of the co-culture provided us with a snapshot of the cellular status at the time of sampling and revealed multi-layered signals of small changes. Thus, in addition to the synthetic connection by sucrose, more links have to be integrated into the mechanistic model of the co-culture. We propose to incorporate the competition for common resources, such as medium components, including citrate and various salts, as we have observed transient uptake or accumulation of citrate, acetate, and ethanol. Furthermore, in both organisms, the ion homeostasis was unbalanced, which might indicate limitations or reduced accessibility of ions through advanced scavenging strategies of the respective co-culture partner. This highlights the requirement for careful medium optimisation in co-cultures in general. The up-regulation of transport processes, particularly for amino acids and degradation of aromatic compounds in *P. putida cscRABY*, suggests the exchange of molecules belonging to these groups. Further studies will be directed to investigate the metabolites in the supernatant to determine whether amino acids or other compounds accumulate. As this phenomenon has been previously reported in growing cultures of *S. elongatus* CCMP 1631^30^ and is commonly observed in marine cyanobacteria consortia^16^. Setting up a precise mechanistic model of co-cultures will contribute to better controllability and stability in multi-species processes and enable upscaling and exploitation for biotechnological applications. However, it is challenging to translate the results of a general grow-associated classification of microbial interactions, e.g., positive/neutral/negative, and the comprehensive results obtained by a multi-OMICs analysis into quantitative, predictive models. Nevertheless, combining phototrophic and heterotrophic organisms holds great potential for co-culture applications, as it combines different metabolic regimes and thus can link CO_2_ fixation to diverse metabolic traits. The ability to efficiently utilise and recycle carbon offers innovative solutions to address environmental and industrial challenges, making these partnerships a promising avenue for future biotechnological advancements. Our findings contribute to a deeper understanding of co-culture dynamics and may, at the end of the day, contribute to harnessing the benefits of synergistic interactions between different microorganisms in biotechnological endeavours.

## Materials and methods

### Strains and culture preparation

The sucrose metabolising strain, *Pseudomonas putida* EM178 *att::Tn7 cscRABY*^14^ harbouring the *cscRABY*-operon was used as the co-culture partner for *Synechococcus elongatus* PCC 7942 *cscB*^10^*. S. elongatus cscB* pre-cultures were first grown in BG11^+^ medium^41^ under continuous illumination of 22 µmol photons s^−1^ m^−2^, 30 °C, and 120 rpm in an orbital shaker (Multitron Pro from Infors HT, Switzerland) without additional aeration. After reaching the stationary phase, the cultures were transferred to BG11^+^ medium supplemented with 150 mM NaCl, inoculated with a 1:20 ratio, and grown under the same conditions. These salt-adapted phototrophic cultures were used for all experiments.

Pre-cultures of *P. putida cscRABY* were grown in 3 mL LB-medium at 30 °C and 220 rpm^14^, and subsequently transferred to a second pre-culture consisting of 3 mL BG11^+^ medium with 3 g L^−1^ sucrose. In the reference experiments, the stationary *P. putida cscRABY* cultures were transferred into BG11^+^ medium supplemented with 150 mM NaCl and 1–3 g L^−1^ sucrose in 100 mL shake flasks and grown under the same conditions as the pre-cultures. The cultures were centrifuged at 4000 × *g* for 5 min. and then resuspended in fresh BG11^+^ supplemented with 150 mM NaCl before being added to the process vessels.

### Physiological investigation of co-culture in 12-well plates (1.6 mL scale)

To investigate potential interactions, experiments were performed in 12-well plates (Brand GmbH, Germany) at a 1.6 mL scale. *S. elongatus cscB* was inoculated to an OD_750_ of 0.05 in BG11^+^ medium supplemented with 150 mM NaCl, and cells were acclimated for two days to salt and other conditions (25–30 °C, 120 rpm, 20 photons µmol m^−2^ s^−2^, incubator Multitron Pro from Infors HT from Switzerland). No additional aeration was provided, and the plates were sealed with laboratory film to prevent water evaporation. Water loss was considered by verifying the volume left in the wells at the end of the experiment. Gene expression of the transporter CscB in cyanobacterial cultures was induced with 0.1 mM Isopropyl ß-D-1-thiogalactopyranoside (IPTG), and an extra amount of sucrose of 1 g L^−1^ was added to support heterotrophic growth at the beginning of the experiment. Co-cultures were started by inoculating different cell counts of *P. putida cscRABY* to achieve different phototroph:heterotroph ratios. For experiments in darkness, the plates were covered in tinfoil.

### Physiological investigation of the co-culture in the membrane reactor

The CellDEG HDC 9.100 Universal Platform (CellDEG GmbH, Germany) consisting of 9 cultivators (HD100 Cultivator) mounted to the platform, an orbital-shaker, and a control unit was used. A partial CO_2_ pressure of 2% and different light profiles for the high-power LED light sources (RX-400 LED light Source from Valoya) were implemented (e.g. constant light of 50 or 120 µmol photons s^−1^ m^−2^ and exponential light t_d_ = 52 h). Cells were grown in BG11^+^ supplemented with 150 mM NaCl and 0.1 mM IPTG for induction of sucrose permease CscB of the phototrophic partner. At the beginning of the process, the pH was set to 7.5, and no further control occurred. The overall volume of each reactor was 95 mL, and water loss through condensation was considered by monitoring the weight of the membrane reactors during the processes. The process was started by inoculating the cyanobacterium from a salt-adapted culture to an OD_750_ of 0.1–0.2 in the cultivation vessel. *P. putida cscRABY* was added to the co-cultures to an OD_600_ of 0.05–0.01. Cell count, optical density, and sucrose concentration were analysed by daily sampling of 1–2 mL of the culture broth.

### Reference experiment with different settings for comparative OMICs analysis

The reference experiment consisted of three different settings in biological triplicates. The setting were the axenic cultures of *S. elongatus cscB*, the axenic culture of *P. putida cscRABY* and the co-cultures, thus 9 samples in total. The experiment started with an acclimatisation phase for the phototrophic partner (Start OD_750_ of 0.1–0.2) under constant light (120 µmol photons s^−1^ m^−2^). After 14–18 h, the co-culture was started by inoculating *P. putida cscRABY* to an OD_600_ of 0.05. A sucrose feed supplied *P. putida cscRABY* axenic cultures with external carbon. Therefore, a cap was designed to enable feeding and in situ sampling from the membrane reactor. The sucrose secreted by *S. elongatus cscB* grown in co-culture was estimated and used to define the sucrose feeding rate for the axenic cultures of *P. putida cscRABY* (see Supplementary Note S5 for the calculation). A batch sucrose of 0.1 g L^−1^ was provided at the beginning, mimicking the initial sucrose production of the phototrophic partner. The axenic cultures of *S. elongatus cscB* were handled as described above. After ~60 h samples for multi-OMICs analysis were taken, centrifuged at 4000 rpm for 5 min (10 mL proteomics) or 13,000 rpm 1 min (1 mL metabolomics and transcriptomics) at 4 °C in a centrifuge 5418R from Eppendorf and subsequently stored at – 80 °C.

### Sample preparation and analytical methods

Samples of the processes were directly used to determine the optical density at 750 nm (600 nm) and cell counts. For further analysis, cells were separated from the medium by centrifugation at 13,000 rpm for 30 s. in a centrifuge 5418R from Eppendorf. Cell counting was carried out as previously described^12^. High-performance liquid chromatography (HPLC) was used to quantify sugars, medium components and common overflow metabolites. The Agilent 1100 series, Waldbronn, Germany with a Shodex SH 1011 column was used for sugar analysis and a Shimadzu LC2030C Plus with a Bio Rad aminex HPX-87H column for other metabolites. The flow rate for the sugar analysis was 0.45 mL min^−1^ with 0.5 mM sulfuric acid, the column was heated to 30 °C, and the refractive index (RI) detector to 50 °C. For analysing medium components and overflow metabolites, a flow rate of 0.6 mL min^−1^ was used with the same aqueous solvent and an RI temperature of 40 °C. Concentrations were calculated by integration of the peak area of each peak and correlation to the corresponding standards.

### Multi OMICs methods

#### Transcriptomics

Samples were sent on dry ice to Eurofins genomic in Konstanz, Germany, for RNA isolation, sequencing, and initial bioinformatic analysis. Results were verified and visualised using the Galaxy platform and R-studio.

#### Metabolomics

Samples were extracted from the cell pellets and separated using two types of columns. A UPLC BEH Amide 2.1 × 100 mm, 1.7 µm analytic column (Waters, Eschborn Germany) with a 400 µL min^−1^ flow rate for hydrophilic interaction liquid chromatography (HILIC) and a Kinetex XB18 2.1 ×100 mm, 1.7 µm (Phenomenex, Aschaffenburg Germany) for reverse phase chromatography (RP) with a 300 µL min^−1^ flow rate. A volume of 5 µL per sample was injected. The autosampler was cooled to 10 °C, and the column oven heated to 40 °C. MS settings in the positive mode were as follows: Gas 1 55 psi, Gas 2 65 psi, Curtain gas 35 psi, temperature 500 °C, Ion Spray Voltage 5500 V, declustering potential 80 V. The mass range of the TOF MS and MS/MS scans were 50–2000 *m/z* and the collision energy was ramped from 15–55 V. MS settings in the negative mode were as follows: Gas 1 55 psi, Gas 2 65 psi, Cur 35 psi, temperature 500 °C, Ion Spray Voltage –4500 V, declustering potential –80 V. The mass range of the TOF MS and MS/MS scans were 50–2000 *m/z* and the collision energy was ramped from –15–55 V. The data was collected in the data-dependent-acquisition mode. A more detailed description of the procedure and data analysis can be found in Supplementary Note S16.

#### Proteomics

Proteins were extracted from cell pellets and trypsin-digested peptide desalting was processed using Bond Elut OMIX C18 tips (Agilent Technologies) following the manufacturer’s instructions. Liquid chromatography-tandem mass spectrometry (LC-MS/MS) proteome analysis was performed using reverse-phase LC on a Dionex Ultimate 3000 RSLC nano 2 system coupled online to a Q Exactive HF mass spectrometer (Thermo Scientific). A more detailed description of the procedure and data analysis can be found in Supplementary Note S16.

### Statistics and reproducibility

All data shown in this work is derived from three biological replicates, i.e. three different cultures that were inoculated from three different precultures, each derived from an individual clone. The mean and the standard deviation were calculated from these three replicates. The 9-fold parallel membrane reactor system allowed to grow all cultures at the same time, reducing variability due to other environmental factors as temperature. The reference experiment was run twice (EI and EII), and data are shown for both experiments (Fig. 3 and Supplementary Note S7). OMICs data is also derived from three biological replicates each, and details on the data analysis are specified in Supplementary Note S16.

### Reporting summary

Further information on research design is available in the Nature Portfolio Reporting Summary linked to this article.

### Supplementary information

Supplementary Information

Description of additional Supplementary Materials

Supplementary Data 1

Supplementary Data 2

Reporting Summary

## Data Availability

The data that supports the findings of this study are available in the supplementary material of this article. All data for transcriptomics and proteomics are presented in the supplementary file Supplementary Data 1 and metabolomics data can be found at MassIVE (https://massive.ucsd.edu) using the data identifier MSV000092369. Source data behind the diagrams and graphs of Figs. 1, 2, and 3 is presented in the supplementary file Supplementary Data 2.
