# Supplementary material for: Pseudomonas putida as saviour for troubled Synechococcus elongatus in a synthetic co-culture – interaction studies based on a multi-OMICs approach
Source: Commun Biol. 2024 Apr 12;7:452. doi: 10.1038/s42003-024-06098-5 (PMC11014904; doi:10.1038/s42003-024-06098-5)
Supplement: Supplementary file 1 — Supplementary Information [file 42003_2024_6098_MOESM1_ESM.pdf]

Supplementary Information

Pseudomonas putida as saviour for troubled *Synechococcus elongatus* in a synthetic co-culture – interaction studies based on a multi-OMICs approach

Franziska Kratzl[1], Marlene Urban[1], Jagroop Pandhal[2], Mengxun Shi[2], Chen Meng[3], Karin Kleigrewe[3], Andreas Kremling[1], Katharina Pflüger-Grau[1]*

[1]Professorship for Systems Biotechnology, TUM School of Engineering and Design, Technical University of Munich, 85748 Garching (Germany)

[2]Department of Chemical and Biological Engineering, University of Sheffield, Sheffield S1 3JD (United Kingdom)

[3]Bavarian Center for Biomolecular Mass Spectrometry (BayBioMS), TUM School of Life Sciences, Technical University of Munich, Freising (Germany)

* For correspondence: Katharina Pflüger-Grau, Professorship for Systems Biotechnology, Technical University of Munich, Boltzmannstr. 15, 85748 Garching (Germany), Tel.: +49 89 289 15765; Fax.: +49 89 289 15766, email: k.pflueger-grau@tum.de

Content

Supplementary Note S1: Phenomenological impact on the co-culture partner's growth at different scales 3

Supplementary Note S2: Influence of illumination and induction time on growth of *S. elongatus cscB* 4

Supplementary Note S3: Effect of external sucrose on *S. elongatus cscB* 4

Supplementary Note S4: Sucrose accumulation in the culture supernatant of *S. elongatus cscB* 5

Supplementary Note S5: Estimation of sucrose feed for *P. putida cscRABY* grown axenically ... 6

Supplementary Note S6: Temperature and light in reference Experiments I and II 7

Supplementary Note S7: Growth rates, optical density, and sucrose in the reference experiments 7

Supplementary Note S8: Cell size and emission at 640-780 nm in EII 8

Supplementary Note S9: Metabolites in the supernatant of experiment II 9

Supplementary Note S10: Carbon fixation and photosynthetic capacity 9

Supplementary Note S11: OMICs data 12

Supplementary Note S12: Translation machinery in *S. elongatus cscB* 16

Supplementary Note S13: Iron limitation 17

Supplementary Note S14: Copper related genes 18

Supplementary Note S15: Stress and Detoxification in *S. elongatus cscB* 19

Supplementary Note S16: Extraction protocols 20

References 22

Supplementary Note S1: Phenomenological impact on the co-culture partner's growth at different scales

The positive effect on *S. elongatus cscB* was not as pronounced in shake flask cultivations. During initial growth, i.e. 22 hours after inoculation, a neutral effect was observed when cell counts normalized to the starting cell counts were compared between the co-culture and the axenic culture. However, this picture changed in the stationary phase (more pronounced after 110 h), where higher cell counts in the axenic culture were observed. However, the presence of the heterotrophic partner in the co-culture resulted in a prolonged pigmentation of the cyanobacterium. These different outcomes between the experiments performed in different scales are not surprising since factors such as mixing and aeration play an important role in the distribution of CO_2 and O_2 in the cultures, which, in turn, also influence bacterial growth and, eventually, cell-cell interaction. Furthermore, the shake flask set-up was used to grow the inoculum of *S. elongatus cscB*, meaning that the cyanobacteria experienced a long adaptation phase (1-2 weeks) to the experimental conditions.

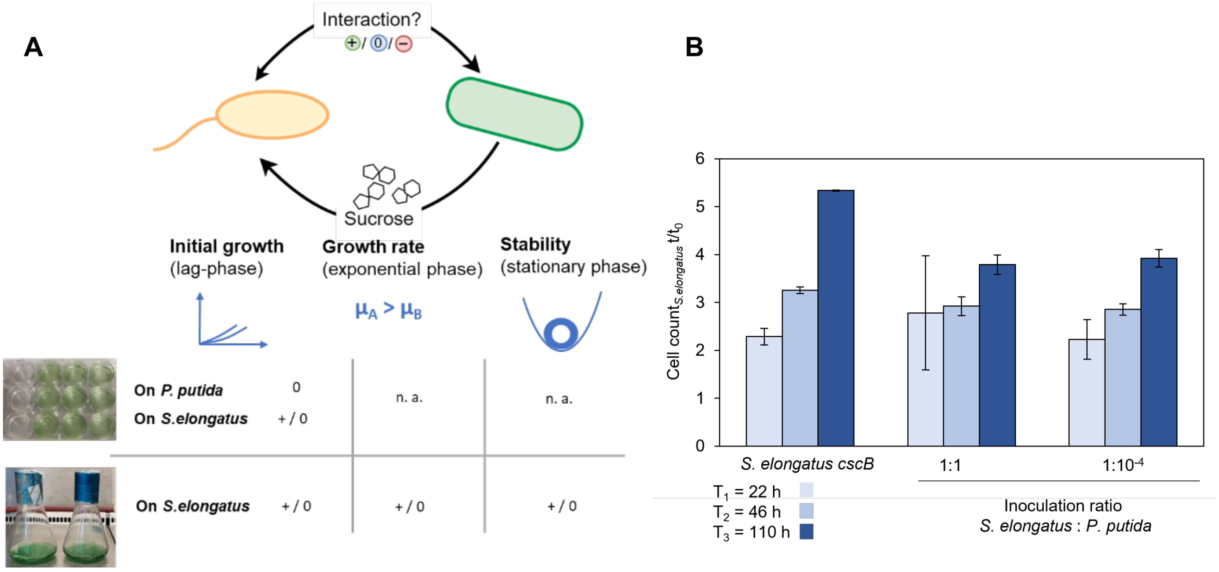

Figure S1: (A) Phenomenological influence of the presence of the co-culture partner on the initial growth, the growth rate, and the long-term stability of the respective partner microbe in different scales. Positive (+) or neutral (0) effects were observed (n. a. = not applicable). **Experimental conditions A**: 12-well plates reaction volume 1.6 mL, shake flasks 15- 20 mL reaction volume. BG11$^+$ supplemented with 150 mM NaCl and 0.1 mM IPTG at 30 °C, 120 rpm in an orbital shaker (Multitron Pro from Infors HT, Switzerland) illuminated with 22 µmol m^{-2} s^{-1}. **(B)** Cell count of *S. elongatus cscB* normed to the inoculum cell count at different time points T_1 = 22 h (initial growth), T_2 = 46 h, and T_3 =110 h of cultivations in shake flasks. **Experimental conditions B**: Shake flasks 20 mL reaction volume. BG11$^+$ supplemented with 150 mM NaCl and 0.1 mM IPTG at 30 °C, 120 rpm in an orbital shaker (Multitron Pro from Infors HT, Switzerland) illuminated with 22 µmol m^{-2} s^{-1}. Standard deviation was calculated from three biological replicates. Two co-cultures with different *S.elongatus:P.putida* inoculation ratios 1:1 and 1:10^{-4} were monitored.

Supplementary Note S2: Influence of illumination and induction time on growth of *S. elongatus cscB*

In Table S1 the growth rates of *S. elongatus cscB* derived from the experiment shown in Figures 2a and 2b of the main manuscript are depicted. The influence of the illumination profile and the time point of induction of *cscB* expression was analysed in the 9-fold parallel membrane reactor system (CellDeg).

Table S1: Growth rate of *S. elongatus cscB* under different illumination conditions in the CellDeg system: Const: with constant illumination of 150 µmol m^{-2} s^{-2} ; Expo: with an exponential light profile. Means and standard deviations were obtained from linear regression from three independent cultures cultivated in parallel.

S. elongatus growth rate, h^{-1}			
Light	Non-induced	Induced, initially	Induced, day 2
Const.	0.064 ± 0.001	0.036 ± 0.001	n.p.[a]
Expo.	0.038 ± 0.004	0.024 ± 0.004	0.040 ± 0.001

[a)]not performed

Supplementary Note S3: Effect of external sucrose on *S. elongatus cscB*

To investigate whether sucrose accumulation within the range of 0 - 3 g L^{-1}, as observed in the co-culture, influenced the growth behaviour of *S. elongatus cscB*, experiments were conducted using varying sucrose concentrations. A growth-reducing effect of sucrose was observed only at very high concentrations of 50 g L^{-1} (resulting in high osmolality, not shown in the data below). It should be noted that when high concentrations of exogenous sucrose are provided, the disaccharide can be taken up by *S. elongatus cscB* when the permease CscB is induced.[1]

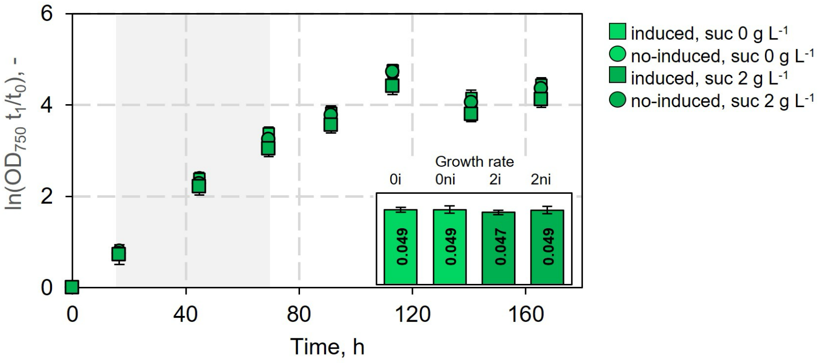

Figure S2: Growth behaviour of *S. elongatus cscB* with and without induction of *cscB* expression in the presence of 2 g L^{-1} sucrose. Control without sucrose addition. **Experimental conditions:** BG11$^+$ supplemented with 150 mM NaCl, in the induced case additionally supplemented with 0.1 mM IPTG. 30 °C, 120 rpm and 22 µmol m^{-2} s^{-1} in a Multitron Pro from Infors HT orbital shaker. 0i = no sucrose, IPTG; 0ni = no sucrose, not IPTG; 2i = 2 g L^{-1} sucrose, IPTG; 2ni = 2 g L^{-1} sucrose, no IPTG.

Supplementary Note S4: Sucrose accumulation in the culture supernatant of *S. elongatus cscB*

In Figure S3 the sucrose accumulated in the culture supernatant of axenically grown *S. elongatus cscB* is shown. Sucrose secretion was initially induced, induced on day 2, or not induced. Cells were grown with an exponential light profile.

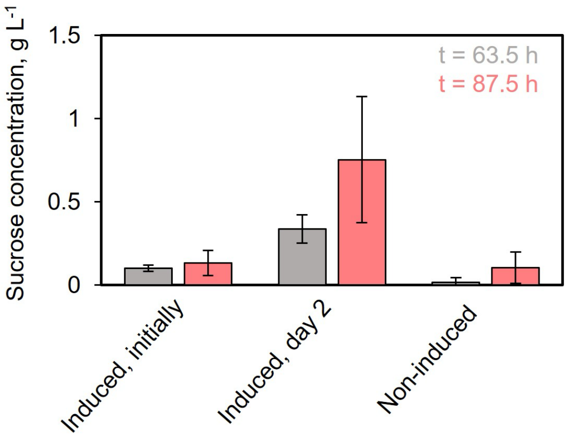

Figure S3: Sucrose accumulation in the culture supernatant of axenically grown *S. elongatus cscB*. **Experimental condition:** BG11$^+$ supplemented with 150 NaCl and 0.1 mM IPTG if culture was induced in the 9-fold membrane reactor system (CellDeg). Exponential light: 24 h 120 µmol m^{-2} s^{-2} for acclimatisation and then exponential rising with a doubling time of t_d = 52 h.

Reference Experiments for OMICs

In this section additional information to the reference experiments are outlined.

Supplementary Note S5: Estimation of sucrose feed for *P. putida cscRABY* grown axenically

In order to obtain a similar growth rate for *P. putida cscRABY* grown axenically or in the co-culture with *S. elongatus cscB*, a sucrose feed was implemented that mimicked the sucrose production of *S. elongatus cscB*. With the correlation (S4.1) between optical density at 600 nm and the cell count C_x for *P. putida,* a connection to the cell dry weight C_{Xcdw} was determined (S4.2):

$$C_x\,[\frac{cell}{mL}] = 2.2\,\cdot\,10^9\,\cdot\,OD_{600} \qquad \text{(S4.1)}$$

$$C_{Xcdw}[\frac{g}{L}] = \frac{C_X}{2.2*10^9}\cdot 0.256 \qquad \text{(S4.2)}$$

With the biomass yield from sucrose of *P. putida cscRABY* of $Y_{Xsuc} = 0.23\frac{g}{g}$, an estimation of the available sucrose C_{suc} was obtained[2]:

$$C_{suc}\,[\frac{g}{L}] = \frac{C_{Xcdw}}{Y_{Xsuc}} \qquad \text{(S4.3)}$$

The sucrose consumption was calculated during exponential growth of *P. putida cscRABY.* After simple linear regression of the cell count of *P. putida cscRABY* within 24 h, a non-specific rate of cells per day of $\frac{Cx}{Time} = 0.192\,\cdot 10^9\,\frac{cells}{mL\cdot d}$ was determined. With this, the respective cell-dry weight per day was calculated and subsequently the metabolised sucrose was estimated. The sucrose feed was kept constant but doubled every 24 h (t_d=24).

$$\frac{OD_{600}}{Time} = \frac{0.192\cdot 10^9\,\frac{cell}{mL\cdot d}}{2.2\cdot\,10^9\,\frac{cell}{mL}} = \,0.22\frac{1}{d}$$

$$\frac{C_{Xcdw}}{Time} = \,0.086\,\frac{1}{d}\cdot 0.256\frac{g}{L} = 0.0221\frac{g}{L\cdot d}$$

$$\frac{C_{suc}}{Time} = \frac{C_{cdw}}{Time}\cdot\frac{1}{Y_{Xsuc}} = \frac{0.0221\frac{g}{L}}{0.23\frac{g}{g}} = 0.0961\,\approx 0.1\frac{g}{L\cdot d}$$

	Sucrose rate, g L^{-1} d^{-1}		
Ref. Experiment	**Day 1**	**Day 2**	**Day 3**
EI	0.1	0.2	0.4
EII	0.065	0.13	0.26

Supplementary Note S6: Temperature and light in reference Experiments I and II

The temperature and the light intensity for reference Experiments I and II are shown in the Figure S4.

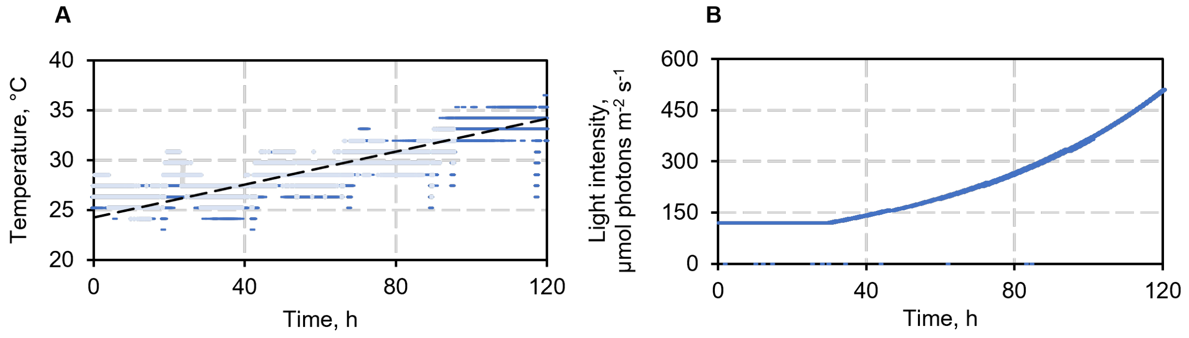

Figure S4: Light and temperature of reference experiments I and II. **A** Temperature profile of reference Experiment I (light blue) and Experiment II (dark blue). **B** light profile of Experiment I and II (no difference).

Supplementary Note S7: Growth rates, optical density, and sucrose in the reference experiments

In Figure S5**A** and **B**, the optical density measured at 750 nm of the co-culture and *S. elongatus cscB* grown axenically in the reference experiments I and II is depicted. Figure S5**C** shows the sucrose accumulation in both reference experiments. Sucrose accumulated to approximately 0.75 g L^{-1} in the co-culture supernatant after 85 h of the process (0.91 g L^{-1} in EII), which suggests that an imbalance between the uptake rate and secretion rate occurs at later stages. It is not possible to differentiate whether this is due to an increase in the sucrose secretion rate of *S. elongatus cscB* or a decrease in the sucrose uptake rate of *P. putida cscRABY*. The high standard deviation of the sucrose accumulation at 85 h is owed to the sucrose concentration in reference Experiment II, where one of the triplicates accumulated significantly more sucrose than the other two cultures. Table S2 shows the growth rates calculated from the reference experiments EI and EII shown in Figure 3 of the main manuscript and Figure S5.

Table S2: Growth rate of *S. elongatus cscB* and *P. putida cscRABY* in axenic and co-cultures in the reference experiments EI and EII. Note that the sucrose feed in axenic cultures of *P. putida cscRABY* resulted in growth rates comparable to those in the co-culture.

	Growth rate, h^{-1}			
	S. elongatus cscB		*P. putida cscRABY*	
	Axenic	Co-culture	Axenic	Co-culture
EI	0.023 ± 0.003	0.067 ± 0.001	0.073 ± 0.001	0.068 ± 0.001
EII	0.030 ± 0.002	0.070 ± 0.002	0.040 ± 0.004	0.043 ± 0.004

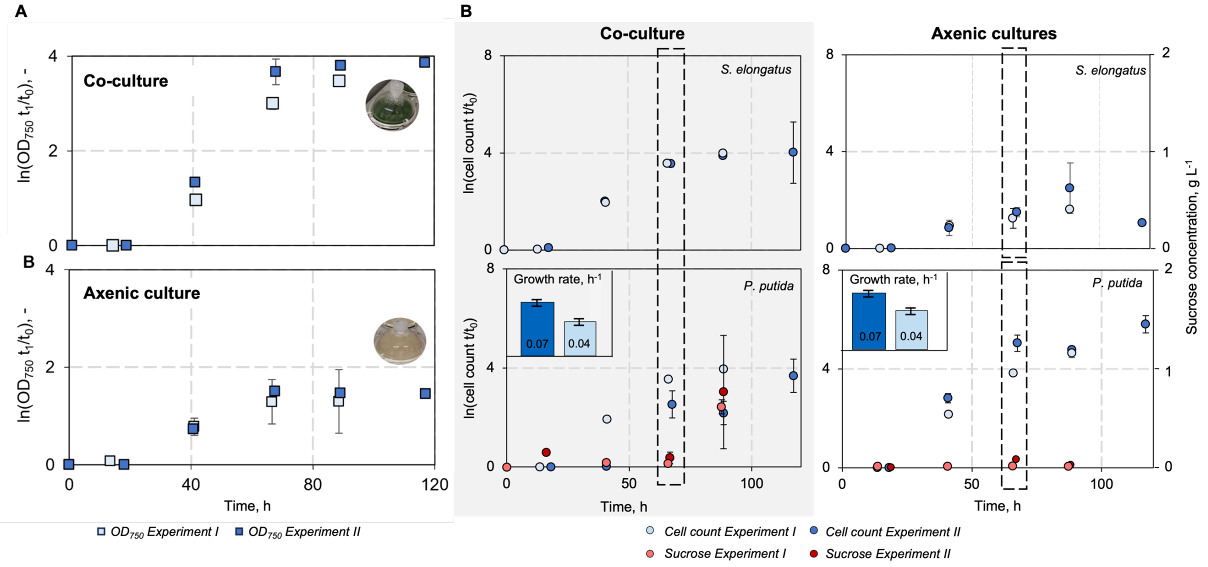

Figure S5: Growth and sucrose concentration in the reference experiments. **(A)** Optical density OD_{750} of the co-culture and *S. elongatus cscB* axenic culture in the reference EI (light blue) and EII (dark blue). A clear photobleaching, loss of chlorophyll, was observed for the *S. elongatus cscB* axenic cultures at the end of the process. The optical density is normalized to the inoculation density of 0.1. Standard deviations derive from biological triplicates. **(B)** Shows the sucrose in both reference Experiments EI and EII.

Supplementary Note S8: Cell size and emission at 640-780 nm in EII

The cell size correlates with the FSC-A (forward scattering), and the emission between 640 - 750 nm corresponds to the emission of pigments such as chlorophyll. The cell size of *S. elongatus cscB* grown in the co-culture and axenically diverges, whereas the cell size of *P. putida cscRABY* remains the same in both cultures. Emission between 640 – 750 nm increases for axenically grown *S. elongatus cscB* and remains constant for cells grown in the co-culture.

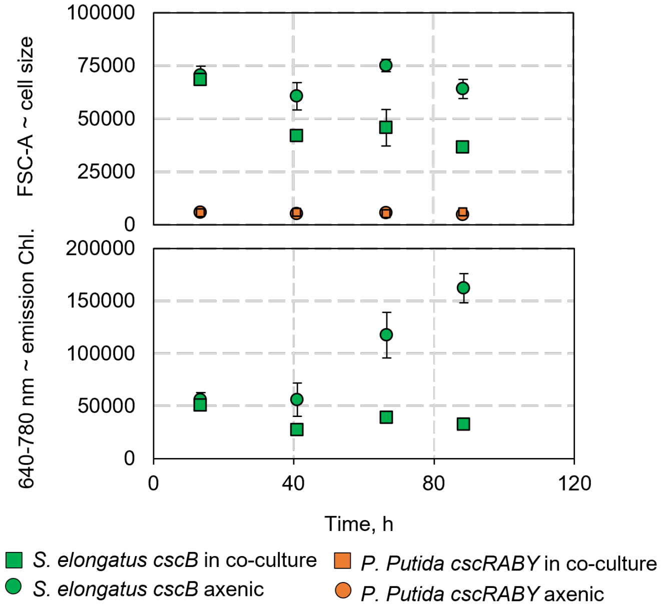

Figure S6: Populations median of FSC-A (forward scatter – area) and fluorescence at 640 – 780 nm measured in the CytoFLEX from Beckman Coulter flow cytometer is shown for the reference Experiment II.

Supplementary Note S9: Metabolites in the supernatant of experiment II

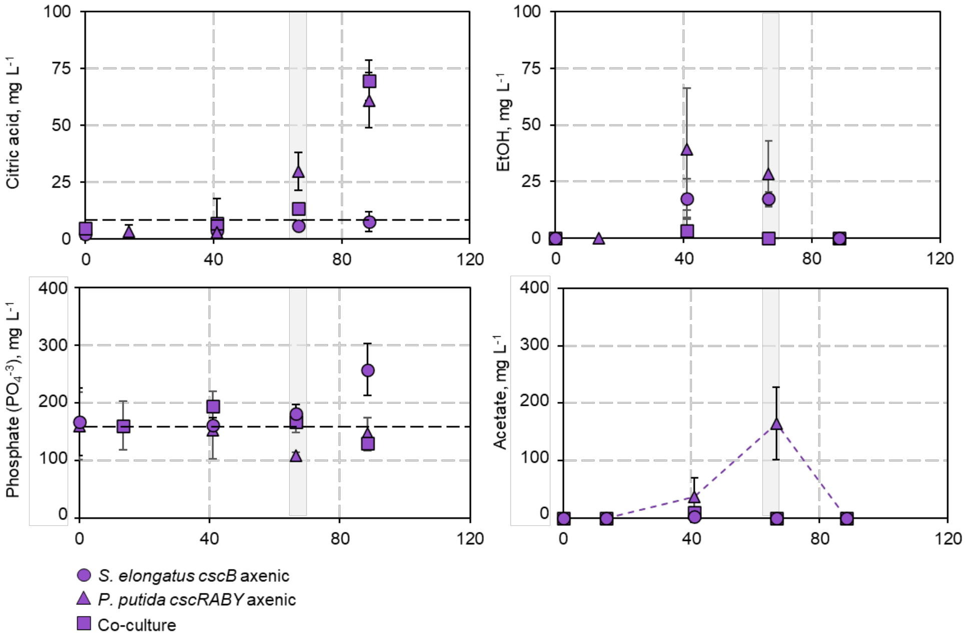

Figure S7: Medium components (Citric acid and phosphate) and overflow metabolites (EtOH and acetate) in the supernatant of experiment II (EII). The dashed black line represents the concentration in the BG11^{+}medium and the grey area represents the sampling points for OMICS. The dashed purple line indicates the course of the acetate concentration in the supernatant of axenically grown *P. putida cscRABY*. Standard deviations are derived from biological triplicates.

Supplementary Note S10: Carbon fixation and photosynthetic capacity

It was reported that the induction of sucrose secretion led to an increased overall CO_2 fixation in *S. elongatus cscB*, as it alleviates the inefficiencies resulting from a limitation in the photosynthetic sink[3,4].

Although the growth rate of the cyanobacterium expressing *cscB* was lower than that of the cells not expressing *cscB*, the total carbon fixed by the cells was higher than under non-inducing conditions (see Figure S8). In the co-culture, the photosynthetic activity of *S. elongatus cscB* seemed to be even higher than in the induced case, as the growth rate surpassed that of the cells grown in non-inducing conditions, and, simultaneously, heterotrophic growth was supported.

The photosynthetic capacity was exemplarily estimated based on the data derived from reference experiment II. Following the induction of the sucrose secreting transporter CscB and inoculation with the co-culture partner an increase in the cyanobacterial growth rate and support of the heterotrophic growth was observed. We assume that the fixed carbon is determined by the sum of the biomass of the cyanobacterium and the sucrose secreted in axenically grown *S. elongatus cscB* cultures expressing *cscB*. For the co-culture, we can expand the term to include the metabolized sucrose, which in turn is converted into biomass of heterotrophic partner.

$$\text{Axenic:} \quad Carbon_{Process} = Carbon_{Biomass} + Carbon_{sucrose}$$
$$\text{Co-culture:} \quad Carbon_{Process} = Carbon_{Biomass} + Carbon_{sucrose} + Carbon_{Putida} \qquad \text{(S8.1)}$$

First, by assuming that one cyanobacterium contains 10^{10} carbons (Bionumbers[1]), we can estimate the amount of carbon that is fixed by producing biomass.

$$Carbon_{Biomass} = cell\ count_{Selongatus} \cdot 10^{10} \quad \text{(S8.2)}$$

Sucrose is a disaccharide composed of glucose and fructose, containing 12 carbon atoms per molecule. With the Avogadro's number N_A, the molar mass of sucrose M_{suc}, and the culture volume V_R the concentration C_{suc} of the sucrose accumulated can be converted into the number of carbon atoms.

$$Carbon_{Sucrose} = \frac{C_{suc} \cdot 12}{M_{suc}} \cdot N_A \cdot V_R \quad \text{(S8.3)}$$

The sucrose necessary for the biomass production of *P. putida cscRABY* can be calculated as described in Equation S6.3.

$$C_{suc} = \frac{C_{Xcdw}}{Y_{Xsuc}}$$

The sucrose concentration can then be converted to the number of carbon atoms with Equation S8.3.

$$Carbon_{putida} = \frac{\frac{C_{Xcdw}}{Y_{Xsuc}} \cdot 12}{M_{suc}} \cdot N_A \cdot V_R$$

As shown in the plot in Figure S8, there is a considerable increase in carbon fixation by the cyanobacterium in the co-culture compared to the axenic culture. It's worth noting that, while the total carbohydrate fixation increased upon adding *P. putida cscRABY*, the sucrose production per cyanobacterial cell decreased.

Table S3: The number of carbon atoms fixed estimated from biomass (grey shaded) and sucrose (blue shaded) per reactor.

		# of carbon from biomass per reactor				# of carbon from sucrose per reactor			
		Axenic cultures		Co-cultures		Axenic cultures		Co-cultures	
Time, h	Co-culture partner	Mean	SD	Mean	SD	Mean	SD	Mean	SD
13.5	*P. putida*			$2.4 \cdot 10^{19}$	$3.0 \cdot 10^{18}$	0	0	0	0
	S.elongatus	$2.3 \cdot 10^{19}$	$2.2 \cdot 10^{18}$	$2.6 \cdot 10^{19}$	$2.7 \cdot 10^{18}$				
41	*P. putida*			$8.1 \cdot 10^{19}$	$7.3 \cdot 10^{18}$	$7.9 \cdot 10^{19}$	$2.7 \cdot 10^{19}$	$1.1 \cdot 10^{20}$	$4.4 \cdot 10^{19}$
	S.elongatus	$6.2 \cdot 10^{19}$	$4.0 \cdot 10^{19}$	$1.9 \cdot 10^{20}$	$1.5 \cdot 10^{19}$				
67	*P. putida*			$3.5 \cdot 10^{20}$	$5.1 \cdot 10^{19}$	$3.9 \cdot 10^{20}$	$6.9 \cdot 10^{19}$	$8.9 \cdot 10^{19}$	$2.0 \cdot 10^{19}$
	S.elongatus	$9.7 \cdot 10^{19}$	$4.0 \cdot 10^{19}$	$9.6 \cdot 10^{20}$	$7.8 \cdot 10^{19}$				
89	*P. putida*			$9.3 \cdot 10^{20}$	$6.0 \cdot 10^{19}$	$7.9 \cdot 10^{20}$	$2.4 \cdot 10^{20}$	$1.3 \cdot 10^{21}$	$1.7 \cdot 10^{20}$
	S.elongatus	$1.1 \cdot 10^{20}$	$5.0 \cdot 10^{19}$	$1.4 \cdot 10^{21}$	$7.3 \cdot 10^{19}$				

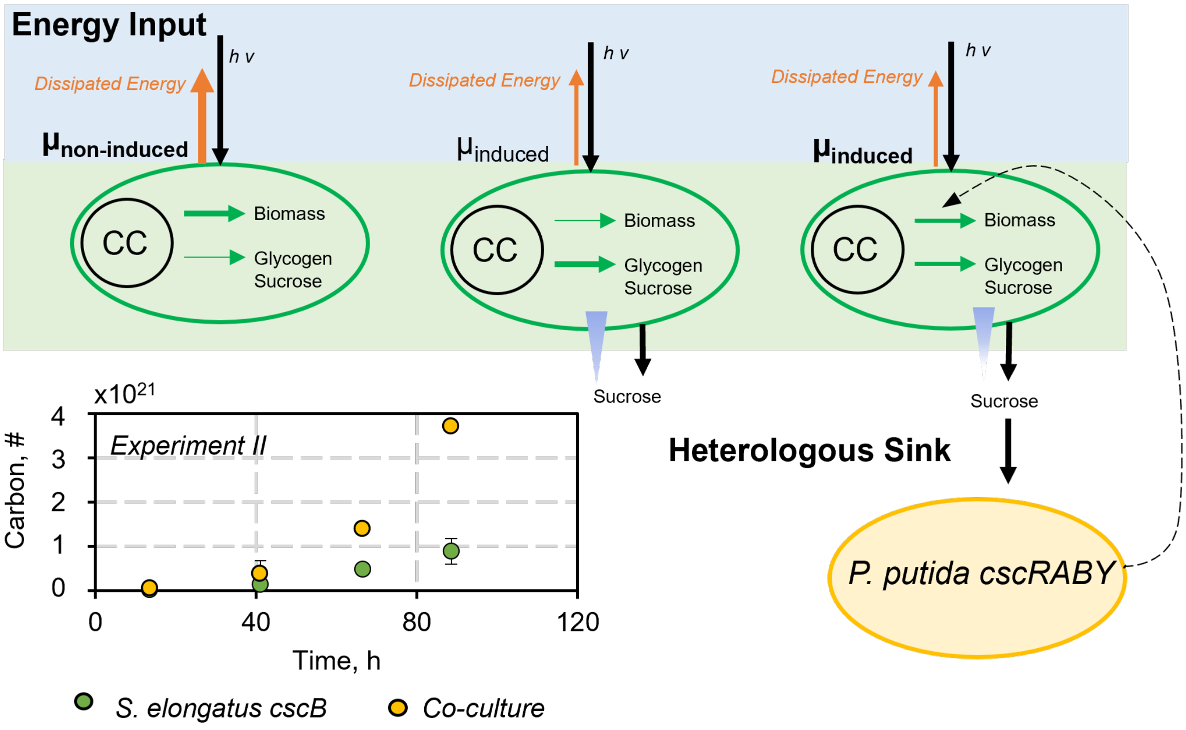

Figure S8: Overview of the energy balance in axenically grown *S. elongatus cscB* under non-inducing and inducing conditions and in co-culture (induced). Energy input from light (h·v, black arrows), is balanced by the energy requirements for cellular metabolism (biomass) and glycogen accumulation/sucrose secretion (green arrows). Excess energy is dissipated through non-photochemical quenching reactions (orange arrows). The amount of energy transfer or carbon flow to each component is visualised by the arrow thickness. Growth rates displayed in bold letters were higher than the growth rate displayed in normal font. The scheme was adapted from Abramson et al.[3]. The diagram illustrates the total carbon fixation (biomass production and sucrose secretion) for axenically and in co-culture grown *S. elongatus cscB* in Experiment II (EII). CC = Calvin cycle, μ = growth rate.

[1]Bionumbers: http://book.bionumbers.org/how-many-photons-does-it-take-to-make-a-cyanobacterium/#:~:text=Given%20that%20it%20requires%20roughly,monomers%20within%20a%20given%20cyanobacterium

Supplementary Note S11: OMICs data

KEGG Orthology (KO) pathway enrichment analysis

To identify overrepresented Kyoto Encyclopedia of Genes and Genomes (KEGG) pathways, we utilised the Bioconductor function *enrichKEEG()* for both co-culture partners. The analysis encompassed all significant DEGs from *P. putida cscRABY* and *S. elongatus cscB,* annotated with ordered locus tags (PP_XX, or SynPCC7942_XX), as these serve as identifiers in KEGG. We applied a cutoff p-value of 0.1 for *P. putida cscRABY*, whereas for *S. elongatus cscB,* the five categories with the lowest p-values are presented.

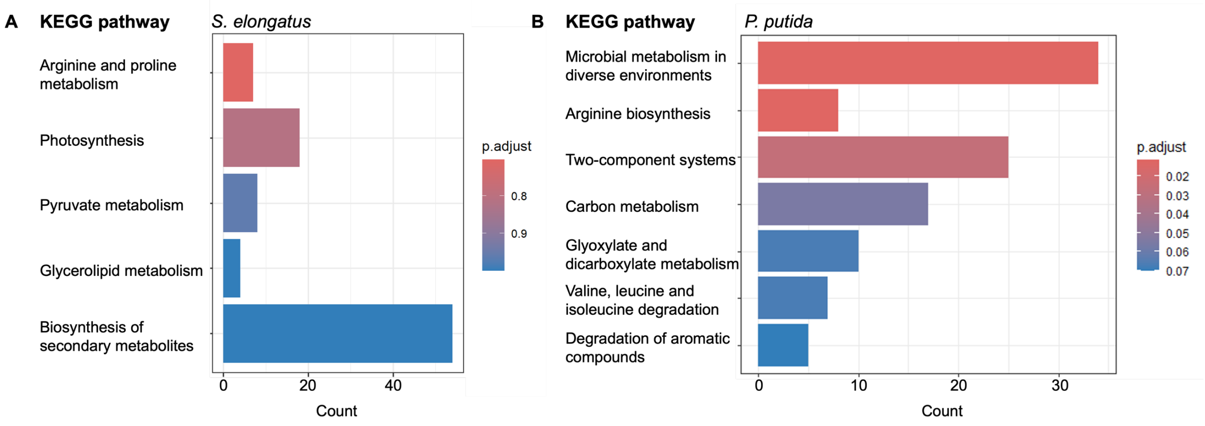

Figure S9: KO enrichment analysis of differentially regulated genes. (**A**) Pathways with the highest adjusted p-values in *S. elongatus cscB* grown in co-culture and (**B**) pathways with enriched gene sets in *P. putida cscRABY* (p.adjust < 0.1) grown in co-culture.

Comparison between Proteomics and Transcriptomics

P. putida cscRABY

Because of the small number of proteins (n_{total} = 232) that were identified comparing the co-culture proteome to that of axenically grown *P. putida cscRABY,* only ~3.6% of the transcriptome find a representative on the proteome level (see Figure S10**A**). This percentage corresponds to 189 proteins that can be linked to a transcript, of which 52% exhibit the same perturbation. This means that they are either less abundant and down-regulated ($log_2(FC) < 0$) or more abundant and up-regulated ($log_2(FC) > 0$). When comparing only the significantly differentially abundant proteins (n_{sig} = 96) with a $|log_2(FC)| > 1.0$ and a p-value < 0.05 to the complete dataset of the transcriptomics, we find that 66 proteins and genes can be linked, with 44% of them exhibiting the same perturbation. When additionally applying the threshold for significantly differentially regulated genes, 10 proteins and genes can be linked, with 20% of them showing a regulation in the same direction.

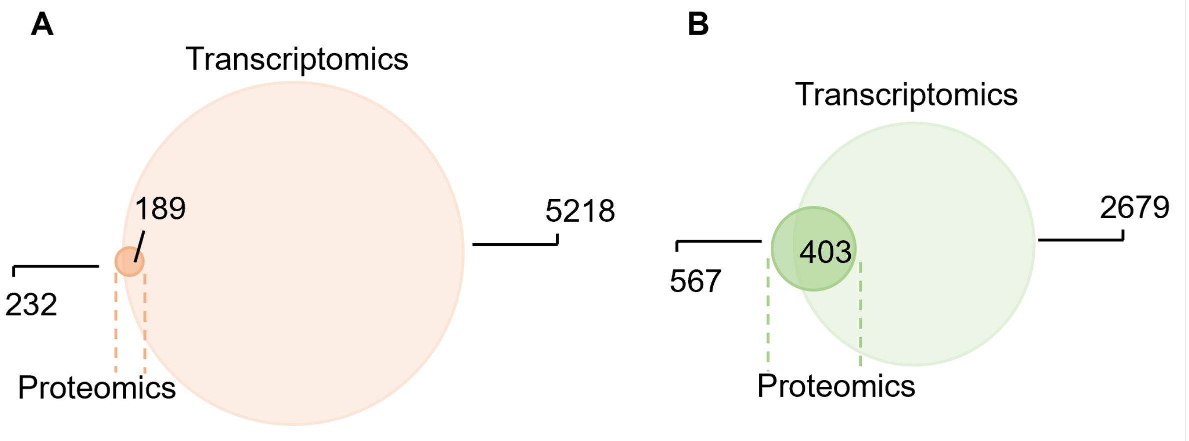

Figure S10: Comparison of the Transcriptome and Proteome of the co-culture compared to axenically grown *P. putida cscRABY* (A) or *S. elongatus cscB* (B).

S. elongatus cscB

Comparing the co-culture proteome to that of axenically grown *S. elongatus cscB*, a total of 567 proteins (n_{total}) were identified. Out of these, 403 proteins could be linked to their respective genes in the transcriptome, which correspond to 7.7% (see Figure S10**B**). Among these gene-protein pairs, 41% exhibit the same perturbation. When comparing the significantly differentially abundant proteins (n_{sig} = 183) with the transcriptomic dataset, 125 proteins can be linked to genes, with 36.8% of them showing the same perturbation. If we expand the filtering criteria to only significantly differentially regulated genes, 42 gene-protein pairs can be found, of which 39% have the same perturbation. It is reported that mRNA transcript abundances only partially correlate with protein abundances, typically explaining approximately one- to two-thirds of the variance in steady-state protein levels[5]. Thus, mRNA levels can provide insights into protein abundance, but other factors, such as post-transcriptional and translational regulation, as well as protein degradation, also contribute to protein abundance.

<u>Vulcano Plots of the metabolites</u>

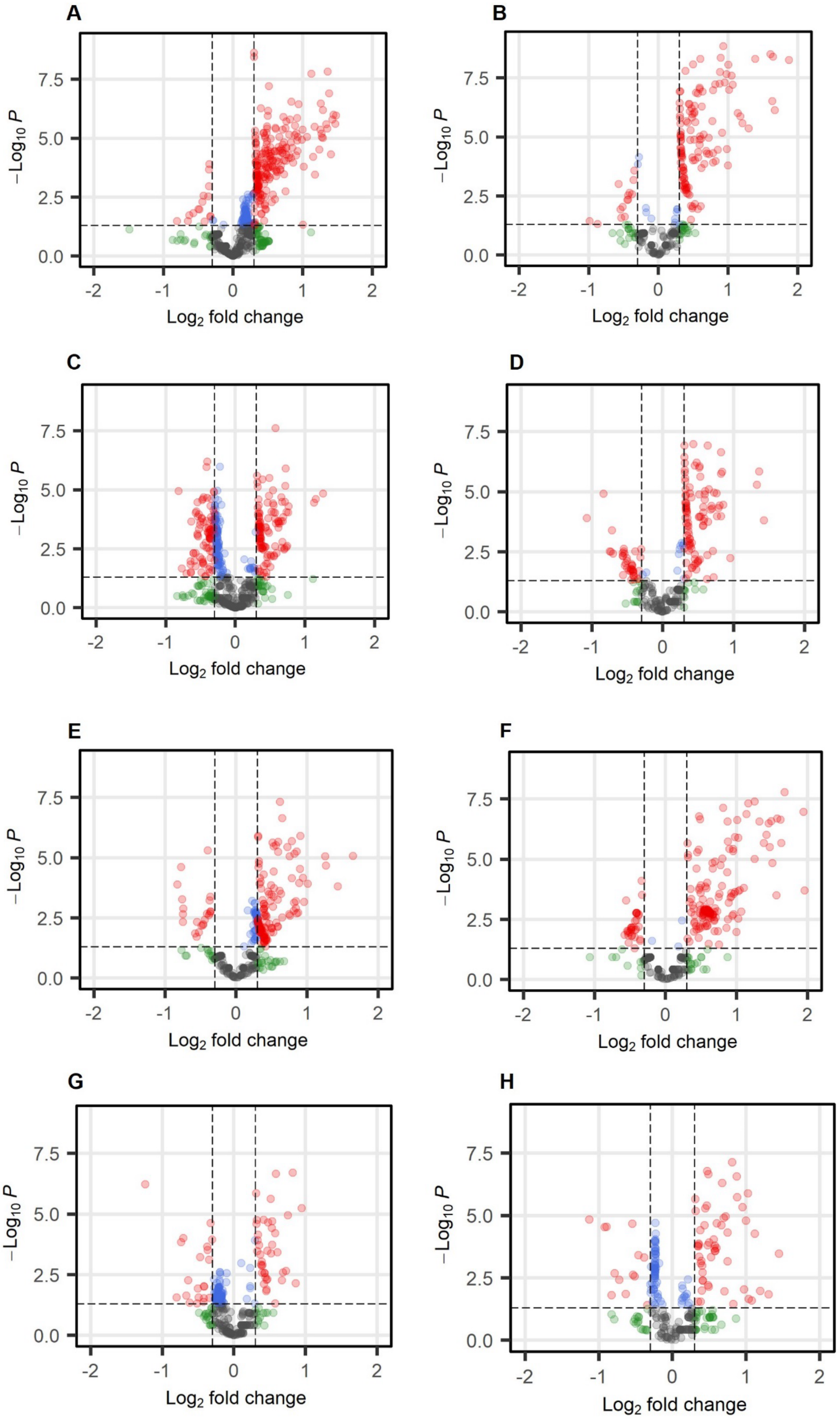

Figure S11: Volcano plots of the metabolites identified with RP-positive MS mode comparing the results obtained in the co-culture to those obtained in the axenic culture of *P. putida cscRABY* **(A)** or *S. elongatus cscB* **(C)**. Volcano plots of the metabolites identified with RP-negative MS mode comparing the results obtained in the co-culture to those obtained in the axenic culture of *P. putida cscRABY* **(D)** or *S. elongatus cscB* **(B).** Volcano plots of the metabolites identified with HILIC-positive MS mode comparing the results obtained in the co-culture to those obtained in the axenic culture of *P. putida cscRABY* **(E)** or *S. elongatus cscB* **(F).** Volcano plots of the metabolites identified with HILIC-negative mode comparing the results obtained in the co-culture to those obtained in the axenic culture of *P. putida cscRABY* **(G)** or *S. elongatus cscB* **(H)**.

Metabolites identified by reference measurements

A

	RP	
	**positive mode**	**negative mode**
LPE 18:1	-0.66	-0.84
AMP	-0.36	--
UMP	--	-0.33
Palmitoleic acid	0.54	--
	**HILIC**	
	**positive mode**	**negative mode**
AMP	-0.37	
Adenine	0.94	0.92
Sucrose	-0.82	--
Glutamine	--	-1.1
	--	--

B

	RP	
	**positive mode**	**negative mode**
Glutamic acid	-0.31	-0.61
Phenylalanine	-0.32	--
Palmitoleic acid	-0.35	-0.31
Trisaccharide	0.43	--
	**HILIC**	
	**positive mode**	**negative mode**
disaccharide	0.74	--
LPE 18:1	--	0.41
UMP	--	0.43
Aspartic acid	--	-1.11
Glutamic acid	--	-0.75
Glutamine	--	-0.88

Figure S12: Shown is the mean difference of metabolites identified by reference measurements that are differentially abundant comparing cells grown in co-culture to those grown in axenic cultures of *S. elongatus cscB* or *P. putida cscRABY*. (**A)** Shows the RP measurements and HILIC measurements in the two different MS modes (+/-) for the co-culture in comparison to *S. elongatus cscB***. (B)** Shows the comparison between the co-culture and *P. putida cscRABY*; The plus or minus indicates the MS-mode.

<u>Supplementary Note S12: Translation machinery in *S. elongatus cscB*</u>

Figure S13 displays the DEGs identified in *S. elongatus cscB* grown in co-cultivation compared to axenically grown cells that belong to the group of genes encoding ribosomes and tRNAs or associated proteins.

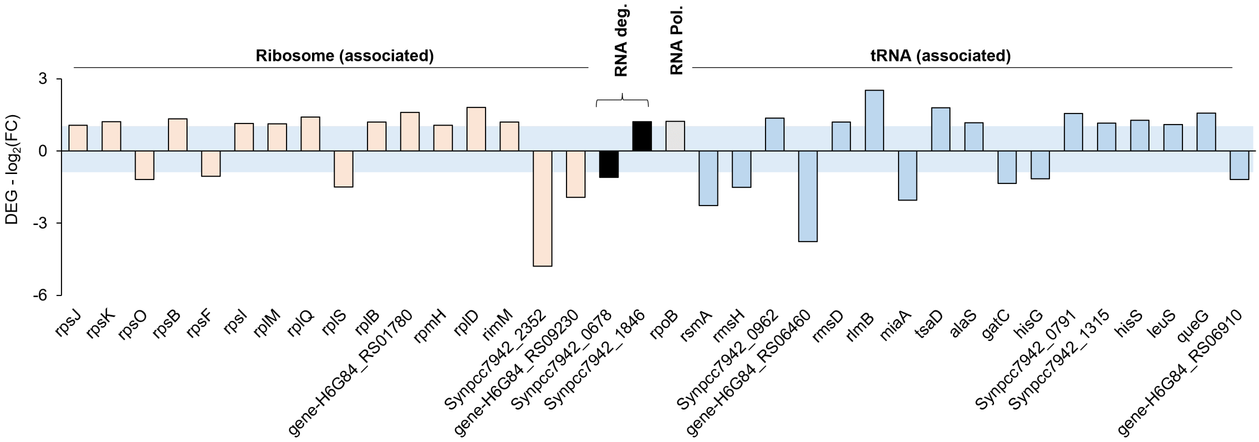

Figure S13: DEGs in *S. elongatus cscB* grouped into ribosome (associated) and tRNA (associated) encoding genes.

Supplementary Note S13: Iron limitation

The effect of elevated and reduced iron concentrations on the growth of *S. elongatus cscB* was investigated in the co-culture and in axenic cultures. Elevated iron concentrations, up to 5 times higher, had no considerable influence on the growth behaviour of the co-culture. Reduction of the iron concentration to 1/100th of the default concentration resulted in a significant decrease in growth of the cyanobacterium.

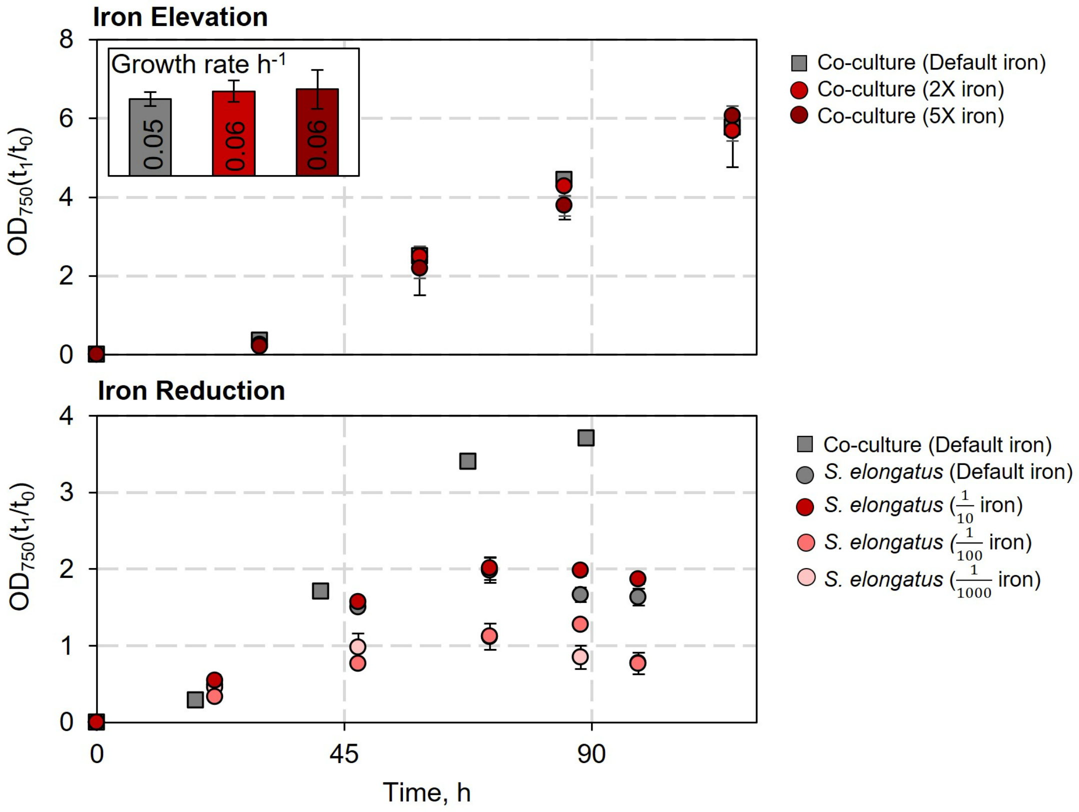

Figure S14: Growth of *S. elongatus cscB* axenically and in co-culture with elevated and reduced iron concentration with an exponential light profile. **Experimental conditions:** 25–30 °C, 2 % CO_2, 95 mL BG11+ supplemented 150 mM NaCl in the CellDeg reactor system, exponential light profile: 24 h constant at 120 µmol m^2 s^1 followed by an exponential rising with a doubling time of t_d = 52 h.

Supplementary Note S14: Copper related genes

Table S4 shows the transcriptional changes of the genes organized in the *cop*/*czc* operon grouped according to their putative function (regulator, outer membrane proteins, or periplasmic proteins). The figure displays a schematic representation of ion detoxification in *P. putida*. The transcript for the copper-sensing metalloregulator CopR-II was up-regulated in the co-culture with a log_2-FC of 2.0. This two-component system is responsible for the activation of transcription of the copper resistance operon *copABCD*. The transcripts for *copA*-I/II (PP_2205 and PP_5380) and *copB*-II (PP_5379) were likewise up-regulated, and in line with this the gene encoding the putative copper receptor OrpC (PP_4838 log_2-FC -2.8) was down-regulated. Genes of the *czc/cus* operon were up-regulated as well. It is located downstream of the *cop* operon and is associated with the cytoplasmic detoxification of copper and silver ions (see Figure S15).

Table S4: Genes of the *cop/czw* operon.

Locus Tag (Gene Name)	Gene function (annotation)	Log_2-FC
**Regulator**		
PP_5383, *copR-II*	Heavy metal response regulator transcription factor	2.0
**Outer Membrane Proteins**		
PP_5385, *czcC*	CzcC family metal RND transporter outer membrane protein	1.4
PP_5379, *cop-II*	Copper resistance protein B	3.0
PP_4828, *orpC*	TonB-dependent copper receptor	-2.8
**Periplasmic Proteins**		
PP_0588, *copZ*	Putative copper-binding chaperone	2.4
PP_2205, *copA-I*	Copper resistance system multicopper oxidase	1.3
PP_5383, *copA-II*	Copper resistance system multicopper oxidase	2.9
PP_5732	Putative metal-binding protein	3.9

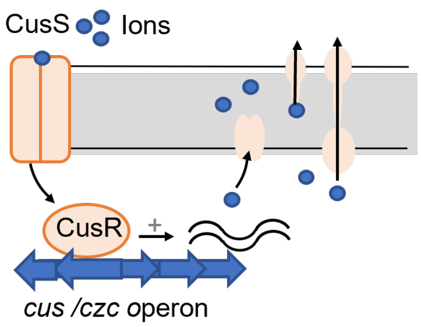

Figure S15: Schematic representation of ion detoxification in *P. putida* cells by the proteins encoded in the *cus/czc*-operon which is regulated by the two-component system CusR/CusS

Supplementary Note S15: Stress and Detoxification in *S. elongatus cscB*

Table S5 displays the proteins that were identified to be differentially abundant in the proteome of co-culture grown cells of *S. elongatus cscB* compared to axenically grown cells that are annotated to be involved in stress and detoxification.

Table S5: Differentially abundant proteins (DAPs) related to stress in *S. elongatus cscB*. Shown are proteins with a log2 FC of >1 and an adjusted p-value of <0.05.

Locus Tag (Gene Name)	Gene function (annotation)	Abundance
Ion related stress proteins		
Synpcc7942_2180	Bacterioferritin comigratory protein	2.0
Synpcc7942_1942	Bacterioferritin comigratory protein-like	2.1
IdiA, Q5N0R0	Iron deficiency-induced protein A	6.0
IrpA, Synpcc7942_1462	Iron-regulated protein A	1.9
Redox related proteins		
Synpcc7942_0109	DNA-binding ferritin-like protein (Oxidative damage protectant)-like	1.2
Synpcc7942_2449	1-Cys peroxiredoxin	2.4
Synpcc7942_0842	Glutathione reductase	1.8
General stress proteins		
Synpcc7942_2468	Putative copper-binding chaperone	-1.2
DpsA, Q9R6T3	DNA protection during starvation protein	-3.6
Synpcc7942_0877	Elongator protein 3/MiaB/NifB	-1.8
FusA, Q31PV4	Elongation factor G	-1.7
Synpcc7942_0297	ATP-dependent zinc metalloprotease FtsH	-1.4
Synpcc7942_0942	ATP-dependent zinc metalloprotease FtsH.1	-1.4
Synpcc7942_0998	ATP-dependent zinc metalloprotease FtsH.3	-1.1

Supplementary Note S16: Extraction protocols

Metabolites extraction:

Metabolomic samples (washed cell pellets) were snap frozen and stored at – 80 °C after centrifugation. Frozen pellets were resuspended in 1 mL of chilled 80% MetOH:H_2O and incubated at -80°C for 20 minutes. After centrifugation (5418 R from Eppendorf) at 14,000 rpm for 5 min. between 4 - 8 °C, the metabolite-containing supernatant was transferred to a tube on dry ice. This process was repeated with 0.5 mL of 80% MetOH, and the supernatant was transferred to the same tube on dry ice. Subsequently, the samples were lyophilized and untargeted analysed using a Nexera UHPLC system (Shimadzu, Duisburg Germany) coupled to a Q-TOF mass spectrometer (TripleTOF 6600 AB Sciex, Darmstadt Germany).

HILIC: A mobile phase of 5 mM ammonium acetate in water (eluent A) and 5 mM ammonium acetate in acetonitrile/water (95/5, v/v) (eluent B). The gradient profile was 100% B from 0 to 1.5 min, 60% B at 8 min and 20% B at 10 min to 11.5 min and 100% B at 12 to 15 min.

RP: Eluent A was 0.1% formic acid and eluent B was 0.1% formic acid in acetonitrile. The gradient profile started with 0.2% B which was held for 0.5 min. Afterwards the concentration of eluent B was increased to 100% until 10 min which was held for 3.25 min. Afterward the column was equilibrated at starting conditions.

Metabolomics Data analysis: The "msconvert" from ProteoWizard [6] was used to convert raw files to mzXML (de-noised by centroid peaks). The bioconductor/R package xcms[7] was used for data processing and feature identification. More specifically, the matchedFilter algorithm was used to identify peaks (full width at half maximum set to 7.5 seconds). Then the peaks were grouped into features using the "peak density" method. The area under the peak was integrated to represent the abundance of features. The retention time was adjusted based on the peak groups present in most samples. To annotate features with names of metabolites, the exact mass and MS2 fragmentation pattern of the measured features were compared to the records in HMBD[8] and the public MS/MS spectra in MSDIAL[9], referred to as MS1 and MS2 annotation, respectively. Missing values were imputed with half of the limit of detection (LOD) methods, i.e., for every feature, the missing values were replaced with half of the minimal measured value of that feature in all measurements.

Protein extraction and digestion:

Protein extraction used a bead-based homogenization method. Cell samples were resuspended in 400 µL lysis buffer (2% SDS, 40mM Tris base, 60mM DTT), frozen at -80 °C overnight, and rapidly thawed at 37 °C for cell breakage. 7 µL of 100x protease inhibitor cocktail (Thermo Fisher Scientific) was added to protect the protein from degradation.

For cell breakage glass beads (~0.5 g) were added to the cell suspension and vigorous vortexed. The resulting crude protein was purified using 2D Clean-Up kit (GE Healthcare). In-solution digestion was conducted to digest the protein for proteomic analysis. 50 µg of protein samples were dissolved in 10 µL urea buffer (8M urea, 100 mM Tris base (pH 8.5), 5mM DTT) and incubated at 37 °C for 30 mins. Then, 1.5 µL of 100 mM iodoacetic acid (IDA) was added to the protein solutions and incubated in the dark at room temperature for 30 mins. Next, 10 µL MS grade trypsin was added in a 1:50 (w/w) protease:protein

ratio to the protein solutions and the solutions were diluted with 58.5 µL 50 mM Tris-HCl (pH 8.5)/ 10 mM $CaCl_2$ to a final concentration of urea of 1M.

The protein solutions were incubated overnight in 37 °C water bath. The trypsin digestion was terminated by adding formic acid to a final concentration of 1%. Samples were dried using a SpeedVac vacuum concentrator (Eppendorf) and stored at -20 °C.

Proteomic Data analysis: Proteomics raw data were processed using MaxQuant (version 2.1.0.0) software using the built-in Andromeda search engine. MaxLFQ algorithm was used for label-free quantification. Carbamidomethyl was chosen as fixed modifications and Acetyl (Protein N-term) and oxidation were chosen as variable modifications. Statistical analysis of protein identification was performed using the LFQ-Analyst website (https://bioinformatics.erc.monash.edu/apps/LFQ-Analyst/).

References

1. Singh, A. K., Santos-Merino, M., Sakkos, J. K., Walker, B. J. & Ducat, D. C. Rubisco regulation in response to altered carbon status in the cyanobacterium Synechococcus elongatus PCC 7942. *Plant Physiol.* **189**, 874–888 (2022).

2. Hobmeier, K., Löwe, H., Liefeldt, S., Kremling, A. & Pflüger-Grau, K. A Nitrate-Blind P. putida Strain Boosts PHA Production in a Synthetic Mixed Culture. *Front. Bioeng. Biotechnol.* **8**, (2020).

3. Abramson, B. W., Kachel, B., Kramer, D. M. & Ducat, D. C. Increased Photochemical Efficiency in Cyanobacteria via an Engineered Sucrose Sink. *Plant Cell Physiol.* **57**, 2451–2460 (2016).

4. Ducat, D. C., Avelar-Rivas, J. A., Way, J. C. & Silver, P. A. Rerouting carbon flux to enhance photosynthetic productivity. *Appl. Environ. Microbiol.* **78**, 2660–2668 (2012).

5. Vogel, C. & Marcotte, E. M. Insights into the regulation of protein abundance from proteomic and transcriptomic analyses. *Nat. Rev. Genet.* **13**, 227–232 (2012).

6. Kessner, D., Chambers, M., Burke, R., Agus, D. & Mallick, P. ProteoWizard: open source software for rapid proteomics tools development. *Bioinformatics* **24**, 2534–2536 (2008).

7. Smith, C. A., Want, E. J., O'Maille, G., Abagyan, R. & Siuzdak, G. XCMS: Processing Mass Spectrometry Data for Metabolite Profiling Using Nonlinear Peak Alignment, Matching, and Identification. *Anal. Chem.* **78**, 779–787 (2006).

8. Wishart, D. S. *et al.* HMDB 4.0: the human metabolome database for 2018. *Nucleic Acids Res.* **46**, D608–D617 (2018).

9. Tsugawa, H. *et al.* MS-DIAL: data-independent MS/MS deconvolution for comprehensive metabolome analysis. *Nat. Methods* **12**, 523–526 (2015).
