# Supplementary material for: Pseudomonas putida as saviour for troubled Synechococcus elongatus in a synthetic co-culture – interaction studies based on a multi-OMICs approach
Source: Commun Biol. 2024 Apr 12;7:452. doi: 10.1038/s42003-024-06098-5 (PMC11014904; doi:10.1038/s42003-024-06098-5)
Supplement: Supplementary file 2 — Description of additional Supplementary Materials [file 42003_2024_6098_MOESM2_ESM.docx]

**Description of Additional Supplementary Files**

**File name:** Supplementary Data 1

**Description:** Results of the multi-OMICs analysis and the source data behind the corresponding graphs in Figures 6, 7, 8, and 9

**File name:** Supplementary Data 2

**Description:** Source Data behind the diagrams and graphs in Figures 1, 2, and 3.
