# Supplementary material for: Pseudomonas putida as saviour for troubled Synechococcus elongatus in a synthetic co-culture – interaction studies based on a multi-OMICs approach
Source: Commun Biol. 2024 Apr 12;7:452. doi: 10.1038/s42003-024-06098-5 (PMC11014904; doi:10.1038/s42003-024-06098-5)
Supplement: Supplementary file 5 — Reporting Summary [file 42003_2024_6098_MOESM5_ESM.pdf]

nature portfolio

Corresponding author(s): Katharina Pflüger-Grau

Last updated by author(s): Feb 27, 2024

Reporting Summary

Nature Portfolio wishes to improve the reproducibility of the work that we publish. This form provides structure for consistency and transparency in reporting. For further information on Nature Portfolio policies, see our Editorial Policies and the Editorial Policy Checklist.

Statistics

For all statistical analyses, confirm that the following items are present in the figure legend, table legend, main text, or Methods section.

n/a	Confirmed	
☐	☒	The exact sample size (*n*) for each experimental group/condition, given as a discrete number and unit of measurement
☐	☒	A statement on whether measurements were taken from distinct samples or whether the same sample was measured repeatedly
☐	☒	The statistical test(s) used AND whether they are one- or two-sided<br>*Only common tests should be described solely by name; describe more complex techniques in the Methods section.*
☒	☐	A description of all covariates tested
☒	☐	A description of any assumptions or corrections, such as tests of normality and adjustment for multiple comparisons
☐	☒	A full description of the statistical parameters including central tendency (e.g. means) or other basic estimates (e.g. regression coefficient) AND variation (e.g. standard deviation) or associated estimates of uncertainty (e.g. confidence intervals)
☒	☐	For null hypothesis testing, the test statistic (e.g. *F*, *t*, *r*) with confidence intervals, effect sizes, degrees of freedom and *P* value noted<br>*Give P values as exact values whenever suitable.*
☒	☐	For Bayesian analysis, information on the choice of priors and Markov chain Monte Carlo settings
☒	☐	For hierarchical and complex designs, identification of the appropriate level for tests and full reporting of outcomes
☒	☐	Estimates of effect sizes (e.g. Cohen's *d*, Pearson's *r*), indicating how they were calculated

Our web collection on statistics for biologists contains articles on many of the points above.

Software and code

Policy information about availability of computer code

Data collection	No software was used for data collection
Data analysis	Metabolomics: The "msconvert" from ProteoWizard was used to convert raw files to mzXML (de-noised by centroid peaks). The bioconductor/R package xcms was used for data processing and feature identification. Proteomics raw data were processed using MaxQuant (version 2.1.0.0) software using the built-in Andromeda search engine. MaxLFQ algorithm was used for label-free quantification.

For manuscripts utilizing custom algorithms or software that are central to the research but not yet described in published literature, software must be made available to editors and reviewers. We strongly encourage code deposition in a community repository (e.g. GitHub). See the Nature Portfolio guidelines for submitting code & software for further information.

Data

Policy information about availability of data

All manuscripts must include a data availability statement. This statement should provide the following information, where applicable:

- Accession codes, unique identifiers, or web links for publicly available datasets
- A description of any restrictions on data availability
- For clinical datasets or third party data, please ensure that the statement adheres to our policy

The data that supports the findings of this study are available in the supplementary material of this article. Fold change data for transcriptomics and proteomics are presented in folder S17-S19 of the Supplementary and metabolomic data can be found at:

http://massive.ucsd.edu/ProteoSAFe/status.jsp?task=be72ac6eeb4f4812a15e82f077b02e7e.
(Once the paper is published, we will make the data publicly available until then, the reviewer can access the data with the link provided. The number for this manuscript is MassIVE MSV000092369)

Research involving human participants, their data, or biological material

Policy information about studies with human participants or human data. See also policy information about sex, gender (identity/presentation), and sexual orientation and race, ethnicity and racism.

Reporting on sex and gender	No research involving human participants, their data or biological material has been performed.
Reporting on race, ethnicity, or other socially relevant groupings	*Please specify the socially constructed or socially relevant categorization variable(s) used in your manuscript and explain why they were used. Please note that such variables should not be used as proxies for other socially constructed/relevant variables (for example, race or ethnicity should not be used as a proxy for socioeconomic status).*<br>*Provide clear definitions of the relevant terms used, how they were provided (by the participants/respondents, the researchers, or third parties), and the method(s) used to classify people into the different categories (e.g. self-report, census or administrative data, social media data, etc.)*<br>*Please provide details about how you controlled for confounding variables in your analyses.*
Population characteristics	*Describe the covariate-relevant population characteristics of the human research participants (e.g. age, genotypic information, past and current diagnosis and treatment categories). If you filled out the behavioural & social sciences study design questions and have nothing to add here, write "See above."*
Recruitment	*Describe how participants were recruited. Outline any potential self-selection bias or other biases that may be present and how these are likely to impact results.*
Ethics oversight	*Identify the organization(s) that approved the study protocol.*

Note that full information on the approval of the study protocol must also be provided in the manuscript.

Field-specific reporting

Please select the one below that is the best fit for your research. If you are not sure, read the appropriate sections before making your selection.

☒ Life sciences ☐ Behavioural & social sciences ☐ Ecological, evolutionary & environmental sciences

For a reference copy of the document with all sections, see nature.com/documents/nr-reporting-summary-flat.pdf

Life sciences study design

All studies must disclose on these points even when the disclosure is negative.

Sample size	Sample size of the reference experiment: Technical triplicates of three different settings (P. putida axenic, S. elongatus axenic and co-culture) Sample triplicates were used for cell count analysis, carbohydrate analysis and omics analysis.
Data exclusions	No data was excluded
Replication	The reference experiment were conducted four times (biological replicates)
Randomization	No randomization was performed. As samples were taken from liquid cultures, the cells entering the analysis were picked randomly anyway.
Blinding	Blinding was not relevant in this study.

Reporting for specific materials, systems and methods

We require information from authors about some types of materials, experimental systems and methods used in many studies. Here, indicate whether each material, system or method listed is relevant to your study. If you are not sure if a list item applies to your research, read the appropriate section before selecting a response.

Materials & experimental systems

n/a	Involved in the study
☒	☐ Antibodies
☒	☐ Eukaryotic cell lines
☒	☐ Palaeontology and archaeology
☒	☐ Animals and other organisms
☒	☐ Clinical data
☒	☐ Dual use research of concern
☒	☐ Plants

Methods

n/a	Involved in the study
☒	☐ ChIP-seq
☐	☒ Flow cytometry
☒	☐ MRI-based neuroimaging

Flow Cytometry

Plots

Confirm that:

☐ The axis labels state the marker and fluorochrome used (e.g. CD4-FITC).

☐ The axis scales are clearly visible. Include numbers along axes only for bottom left plot of group (a 'group' is an analysis of identical markers).

☐ All plots are contour plots with outliers or pseudocolor plots.

☐ A numerical value for number of cells or percentage (with statistics) is provided.

Methodology

Sample preparation	Simple dilution of the bacterial cells and addition of a fluorescence dye N-(3-Triethylammoniumpropyl)-4-(4-(4-(diethylamino)phenyl)butadienyl)pyridinium dibromide (see M&M for details).
Instrument	Beckman coulter flow cytometer was used (Cytoflex) The device is equipped with two lasers (488 nm and 638 nm) and eight fluorescence channels.
Software	CytoFlex Acquisiton and Analysis Software
Cell population abundance	Samples were used to count the cell count of the cell population in the co-culture. Dilutions were adjusted to obtain reasonable cell count in the gates set for the populations. (Usually between 20 - 1000 cells)
Gating strategy	Cells were distinguished from noise due to size and due to the fluorescence dye (SSC-H gate). Furhtermore, distinct emission of the cells P. putida (488-585 nm) and S. elongatus (640-660 nm) were used to gate the populations.

☐ Tick this box to confirm that a figure exemplifying the gating strategy is provided in the Supplementary Information.
